# *Mycobacterium tuberculosis*-triggered Hippo pathway orchestrates CXCL1/2 expression to modulate host immune responses

**DOI:** 10.1038/srep37695

**Published:** 2016-11-24

**Authors:** Monoranjan Boro, Vikas Singh, Kithiganahalli Narayanaswamy Balaji

**Affiliations:** 1 Department of Microbiology and Cell Biology, Indian Institute of Science, Bangalore 560012, Karnataka, India

## Abstract

*Mycobacterium tuberculosis* (Mtb) pathogenesis encompasses a plethora of finely regulated alterations within the host which eventually coin the outcome of infection. Chemokines are important components in directing immune cell recruitment to the site of infection, and shaping the disease progression. Here, we demonstrate that Hippo (mammalian sterile 20–like 1 and 2 kinases, MST1/2, in mammals), is activated during mycobacterial infection in a toll-like receptor (TLR) 2-interleukin receptor-1 associated kinases (IRAK1/4)-dependent manner. Mtb-triggered Hippo signaling modulates the expression and secretion of chemokines (CXCL1 and CXCL2); as silencing MST1/2 compromised the ability of Mtb to furnish the same. Further insight into the mechanism of Hippo-mediated regulation of chemokines revealed the role for a non-canonical Hippo effector interferon (IFN) regulatory factor (IRF) 3 in the process and marked the effect to be independent of LATS1. Alongside their ability to guide directed recruitment of immune cells, we have uncovered a paracrine role for Hippo-mediated secretion of CXCL1 and CXCL2 in the production of anti-microbial peptides (beta-defensins), iNOS, NOX2 and pro-inflammatory molecules during mycobacterial infection of the host. This study highlights the involvement of TLR2-IRAK1/4-MST1/2-IRF3 axis in Mtb-triggered modulation of chemokines and identifies Hippo signaling as a novel regulator of host-mycobacterial interactions.

Tuberculosis (TB), caused by *Mycobacterium tuberculosis* (Mtb), has successfully persisted in the human population and continues to be of immense medical concern due to its ability to tactfully modulate its host^1^,^2^. Interaction of the host with mycobacteria initiates a series of innate and adaptive immune responses to contain the infection. Macrophages, the sentinels of innate immune system serve as the primary niche to the pathogen and mediate the early retaliation^3^. In this regard, chemokines produced by immune cells have been implicated in controlling key processes during mycobacterial infection and in defining the concerted recruitment of various other immune cells like neutrophils, T- and B-lymphocytes to curtail the spread of the pathogen^4^,^5^,^6^,^7^,^8^.

The delicate balance in the action of chemokines is cardinal in dictating the outcome of mycobacterial infection. Of the several chemokines identified, some are constitutive and others are induced in presence of inflammatory stimuli. The production of chemokines such as MCP1, MIP1α, MIP1β, RANTES and MIP2 has been well documented during mycobacterial infection *in vitro* as well as *in vivo* in bronchoalveolar lavage of TB-stricken patients^9^ and some of these are used as biomarkers for TB progression^10^. Efforts ensue to study the role of individual chemokines but it is strenuous to achieve due to a significant level of functional redundancy. For instance, MCP1^−/−^ mice have been reported to show increased mycobacterial burden in lungs and spleen three weeks post-infection, however, the effect is normalized to CCR2^−/−^ mice at later stages. Also, the knockouts for some chemokines fail to show any detectable phenotype. Therefore, it is plausible that the study of the regulatory mechanisms for these molecules may advance the purpose^11^. The modulation of the intricate host signaling networks majorly governs the net observable responses. In this regard, it has been reported that alongside immunologically active pathways, many early development-specific signaling cascades are reprogrammed to furnish infection-defined defensive measures^12^,^13^,^14^,^15^,^16^,^17^,^18^,^19^. One such pathway which is garnering attention is Hippo signaling pathway.

The core components of the Hippo pathway include two serine threonine kinases, MST1/2 (Hippo in Drosophila) and LATS1/2; and two transcriptional coactivators YAP and TAZ^20^. Signaling initiates by phosphorylation-mediated activation of MST1/2 sequentially followed by MST1/2-dependent phosphorylation of LATS1/2 and MOB1. Activated LATS1/2 then phosphorylate YAP and TAZ, promoting their cytoplasmic sequestration and eventual degradation. Intranuclear YAP/TAZ interact with TEAD transcription factors to bring about the expression of target genes^21^,^22^,^23^. Originally identified in Drosophila to promote apoptosis^24^, inhibit cell proliferation and tumorigenesis^23^,^25^; Hippo signaling components are now being implicated in T-cell deficiency associated with viral, bacterial and fungal infections^26^,^27^,^28^,^29^ and also in autoimmune manifestations^30^. MST1/2 deficient mice were reported to have hypoplastic lymphoid organs because of defective T cell migration^30^,^31^. It was also shown that MST2 deficient mice produce decreased levels of proinflammatory cytokines and chemokine CCL2 during the process of retinal detachment^32^. Recent studies demonstrate the activation of MST1 in human dendritic cells by chemokines CCL19 and CCL21 to regulate trafficking of dendritic cells^33^. Also to our interest, recently Geng *et al*. reported the activation of MST1 and MST2 by cell surface TLRs via a MyD88-dependent pathway, leading to enhanced ROS production and bacterial clearance^34^. However, the status of Hippo pathway during mycobacterial infection of macrophages remains obscure.

In view of the available literature background, we set out to understand the status of Hippo pathway during mycobacterial infection and its implication in steering the production of chemokines during the pathogenesis of Mtb. The present study demonstrates that pathogen-specific TLR2-mediated activation of Hippo signaling in macrophages through IRAK1 and IRAK4 renders MST1/2 to be a regulatory node in the modulation of chemokines, CXCL1 and CXCL2, through IRF3; and identifies these chemokines as novel paracrine regulators for the production of anti-microbial molecules and inflammatory mediators during mycobacterial infection of the host.

## Results

### *Mycobacterium tuberculosis* (Mtb)-driven TLR2 signaling regulates Hippo pathway

**Figure 1:**
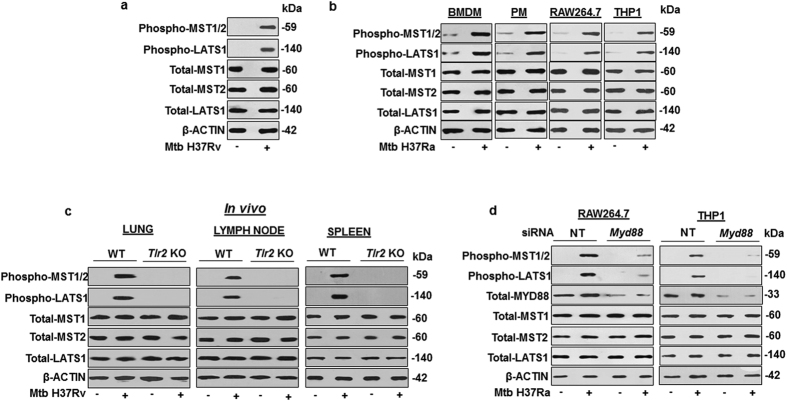
*Mycobacterium tuberculosis* (Mtb)-driven TLR2 signaling activates Hippo pathway. (**a**) Mouse primary peritoneal macrophages were infected with Mtb H37Rv for 1 h to assess the phosphorylated forms of MST1/2, LATS1 and the total levels of MST1, MST2 and LATS1 by immunoblotting. (**b**) Hippo pathway activation was assessed by immunoblotting in BMDM, PM, RAW264.7 and THP1 macrophages infected with Mtb H37Ra for 1 h. (**c**) Hippo pathway was assessed in lung, lymph node and spleen of WT and *Tlr2* KO mice infected with Mtb H37Rv by aerosol route. (**d**) *Myd88* was knocked down in RAW264.7 and THP1 macrophages followed by infection with Mtb H37Ra for 1 h to check the status of Hippo pathway by immunoblotting. β-ACTIN was used as loading control for immunoblotting experiments on total cell lysates. Blots are representatives of three independent experiments. siRNA, small interfering RNA; WT, wild type; NT, non-targeting; BMDM, bone marrow derived macrophages; PM, peritoneal macrophages; THP1, human leukemia monocytic cell line; h, hour; KO, knock out.

Cell fate decisions modulating immunological homeostasis during pathogenic mycobacterial infections often play critical role in overall outcome of the infection. In this perspective, the Hippo pathway, a key regulator of immunological homeostasis appears to influence significant steps during initiation or progression of various patho-physiological conditions^31^. Interestingly, role of Hippo signaling during mycobacterial infection of the host immune cells and subsequent manifestations towards disease progression is not known. In this regard, we found activation of Hippo signaling in Mtb H37Rv infected mouse peritoneal macrophages (PM) (Fig. 1a) as evaluated by increase in the phosphorylation status of canonical Hippo pathway components; MST1/2, LATS1. However, the total pool of MST1, MST2 and LATS1 did not alter during mycobacterial infection as indicated in (Fig. 1a). Hippo pathway activation by Mtb was also confirmed in bone marrow derived macrophages (BMDM), RAW264.7 and THP1 macrophages (Fig. 1b). Induced phosphorylation of MST1/2 and LATS1 clearly suggest Hippo pathway activation in Mtb-infected macrophages. A recent report suggests the ability of TLR1, TLR2 and TLR4 to substantially activate MST1 and MST2 when treated with specific TLR agonists^34^. Further, earlier understanding from our and others’ work indicate that of all other PRRs, mycobacteria-specific responsiveness of TLR2 results in the vigorous activation of various signaling pathways in macrophages^12^,^13^,^14^,^35^. In this perspective, the ability of Mtb to activate effectors of Hippo signaling was significantly compromised in lungs, spleen and lymph node of *tlr2* knockout mice (Fig. 1c). Altogether, the above results suggest an essential role for Mtb specific TLR2 signaling in regulating Hippo pathway activation.

### IRAK1 and IRAK4 form an essential link in the pathogen-specific activation of Hippo signaling through TLR2

**Figure 2:**
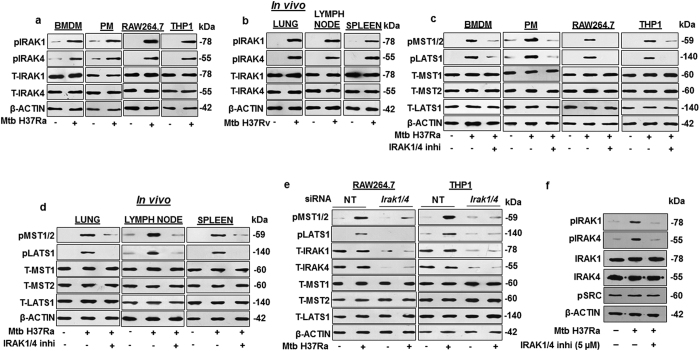
*Mycobacterium tuberculosis* activated IRAK1 and IRAK4 drive Hippo pathway activation. (**a**) Activation of IRAK1 and IRAK4 were assessed in BMDM, PM, RAW264.7 and THP1 macrophages infected with Mtb H37Ra for 1 h by immunoblotting. (**b**) *In vivo* analysis of IRAK1 and IRAK4 activation in lung, lymph node and spleen of mice infected with Mtb H37Rv by aerosol route. (**c**) IRAK1 and IRAK4 were inhibited in BMDM, PM, RAW264.7 and THP1 macrophages by IRAK1/4 inhibitor to assess their effect on Hippo pathway activation by Mtb. (**d**) Mice were intra-venously injected with IRAK1/4 inhibitor followed by infection with Mtb H37Ra to assess the Hippo pathway activation in spleen, lung and lymph node. (**e**) IRAK1/4 were knocked down in RAW264.7 and THP1 macrophages by *Irak1/4* siRNA followed by infection with Mtb for 1 h to assess the Hippo pathway. (**f**) IRAK1/4 inhibitor specificity was assessed by activation of IRAK1, IRAK4 and pSRC by immunoblotting. Blots are representatives of three independent experiments. p, phospho; T, total; BMDM, bone marrow derived macrophages; PM, peritoneal macrophages; h, hour; siRNA, small interfering RNA; NT, non-targeting; inhi, inhibitor. THP1, human leukemia monocytic cell line.

Signal transduction through TLR2 involves the interplay of key TLR adaptors like MyD88, IRAK1, IRAK2, IRAK4 and IRAKM^36^,^37^. Upon activation of TLR, IRAK4 undergoes autophosphorylation which then phosphorylate and activate IRAK1. Hence, the status of IRAK1 and IRAK4 were first assessed in Mtb infected macrophages. IRAK1 and IRAK4 were found to be activated upon Mtb infection, as evaluated by increase in the phosphorylated forms of the concerned adaptors, both *in vitro* (Fig. 2a) and *in vivo* (Fig. 2b). It was recently demonstrated that TLR-dependent activation of MST1 and MST2 requires MyD88^34^, which could be observed in our system as well (Fig. 1d); however, the possible downstream effectors of MyD88 were hitherto unidentified. In this regard, inhibition of IRAK1 and IRAK4 in BMDM, PM, RAW264.7 and THP1 macrophages compromised the ability of Mtb to induce Hippo pathway activation (Fig. 2c). Further, inhibition of IRAK1 and IRAK4 *in vivo* (Fig. 2d) also reduced Mtb-induced activation of Hippo signaling in mouse lung, lymph node and spleen. The positive role of IRAK1 and IRAK4 in the regulation of Hippo pathway was further validated by knocking down IRAK1 and IRAK4 (Fig. 2e) by utilizing specific *Irak1/4* siRNA in RAW264.7 and THP1 macrophages, which significantly reduced the level of Mtb-induced Hippo pathway activation. Specificity of IRAK1/4 inhibitor has been validated (Fig. 2f). Altogether, these results clearly demonstrate that kinases IRAK1 and IRAK4 are novel upstream positive regulators of Hippo pathway in Mtb-infected macrophages.

### Mtb activated IRAK1 and IRAK4 directly interact with MST1/2

**Figure 3:**
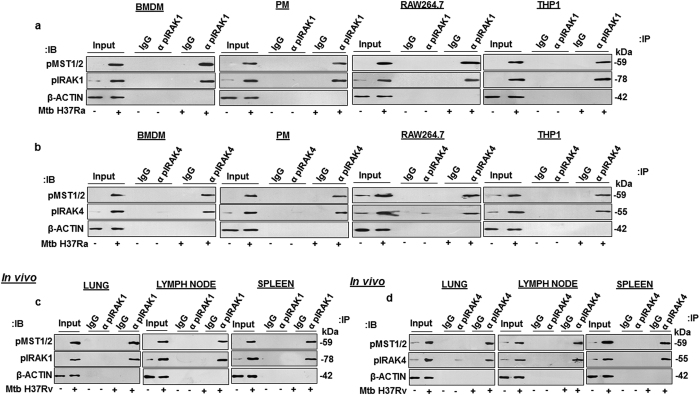
*Mycobacterium tuberculosis* activated IRAK1 and IRAK4 interact with MST1/2. (**a,b**) Immuno-pulldown experiments were performed with anti pIRAK1 (**a**) or anti pIRAK4 (**b**) antibody in Mtb-infected or uninfected BMDM, PM, RAW264.7 and THP1 macrophages to assess the interaction of pIRAK1 or pIRAK4 with phospho MST1/2. (**c**,**d**) Immuno-pulldown experiments were performed with anti pIRAK1 (**c**) or anti pIRAK4 (**d**) in lung, spleen and lymph node of mice infected with Mtb H37Rv. BMDM, bone marrow derived macrophages; PM, peritoneal macrophages; THP1, human leukemia monocytic cell line; p, phospho; IgG, immunoglobulin G; IB, immunoblotting; IP, immuno-pulldown; α, anti.

Having validated the role for IRAK1 and IRAK4 in modulating Hippo pathway, the mechanisms underlying IRAK1- and IRAK4- mediated MST1/2 activation during mycobacterial infection of macrophages was investigated. Since, mycobacterial infection of macrophages vigorously increases the activating phosphorylation of IRAK1, IRAK4 and MST1/2; therefore, immuno-pulldown experiments were performed with anti phospho-IRAK1 (Fig. 3a) or anti phospho-IRAK4 antibody (Fig. 3b) and analyzed for phospho-MST1/2 in Mtb-infected BMDM, PM, RAW264.7 and THP1 macrophages. Importantly, phospho-MST1/2 was identified as the interacting partner for phospho-IRAK1 and phospho-IRAK4 (Fig. 3a,b) across different macrophage types during mycobacterial infection. Consistent with these results, Mtb aerosolized mouse’s lung, lymph node and spleen clearly showed the Mtb-induced interaction of phospho-IRAK1 or phospho-IRAK4 with phospho-MST1/2 (Fig. 3c,d). Altogether, these results clearly demonstrate that MST1/2 are novel interacting partners of IRAK1 and IRAK4 in Mtb-infected macrophages.

### Mycobacteria-induced-TLR2-driven Hippo signaling modulates the expression of chemokines CXCL1 and CXCL2

**Figure 4:**
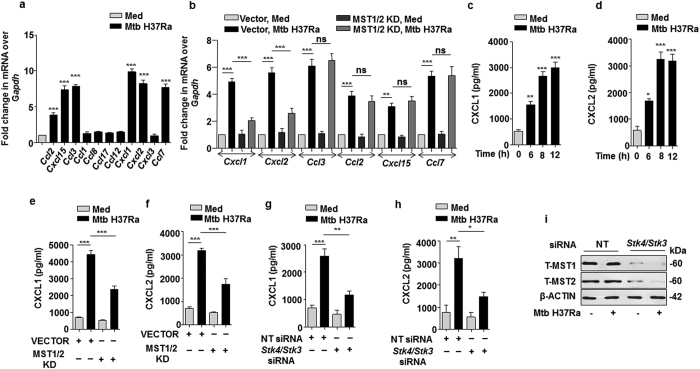
MST1/2 are involved in the production of CXCL1 and CXCL2 upon mycobacterial infection of macrophages. (**a**) Murine peritoneal macrophages were infected with Mtb for 12 h and the transcript levels of the indicated chemokines were assessed by qRT-PCR. (**b**) RAW264.7 macrophages were transfected with MST1/2 KD constructs for 36 h followed by infection with Mtb for 12 h to analyze the transcript levels of the indicated chemokines by qRT-PCR. (**c** and **d**) Macrophages were infected with Mtb for the indicated time points to detect the levels of CXCL1 (**c**) or CXCL2 (**d**) in culture supernatants by ELISA. (**e**–**h**) Macrophages were transfected with MST1/2 KD constructs (**e**,**f**) or *Stk4/Stk3* siRNAs (**g** and **h**) for 36 h followed by infection with Mtb for 12 h and culture supernatants were analyzed for CXCL1 (**e**,**g**) and CXCL2 (**f**,**h**) by ELISA. (**i**) Validation of MST1/2 knocked down in RAW264.7 macrophages by immunoblotting. All the data represent mean ± SE (N = 3), *p < 0.05, **p < 0.005, ***p < 0.0001 (One way Anova or t test). KD, kinase dead; Med, medium; pg, picogram; siRNA, small interfering RNA; h, hour; ns, not significant; T, total; NT, non-targeting.

Chemokines serve critical function in directing immune cells to inflammatory site; which might be decisive in controlling the severity of disease^38^,^39^. In this regard, it is important to study the regulatory mechanisms involved in the production of chemokines. A screen for 11 inflammatory chemokines^9^ identified CCL2, CCL3, CCL7, CXCL1, CXCL2 and CXCL15 to be induced upon mycobacterial infection (Fig. 4a). Further, the possible role of Hippo signaling in regulating Mtb-induced chemokines expression was implicated by over expressing kinase dead forms of MST1/2 in RAW264.7 macrophages. This marked CXCL1 and CXCL2 to be MST1/2-responsive as Mtb failed to induce these chemokines in presence of catalytically inactive MST1/2 (Fig. 4b). In agreement with the above observations, it was found that there was infection-induced secretion of CXCL1 and CXCL2 into the culture supernatants (Fig. 4c,d), which was compromised in the presence of MST1/2 KD constructs (Fig. 4e,f) or *Stk4*/*Stk3* siRNA (Fig. 4g,h). The knock down efficiency of MST1 and MST2 by *Stk4*/*Stk3* siRNA is shown in Fig. 4i. These observations clearly revealed the novel role of MST1/2 in Mtb-induced production of CXCL1 and CXCL2.

### MST1/2-regulated production of chemokines is independent of LATS1

**Figure 5:**
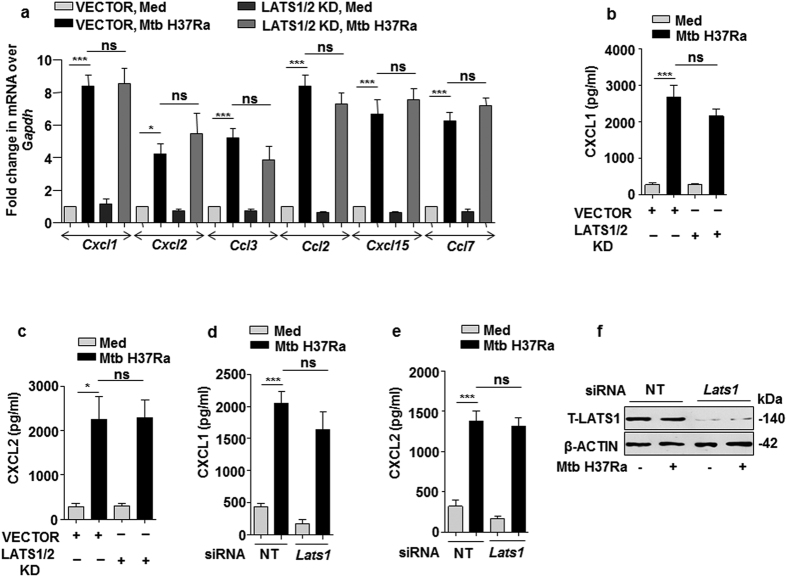
LATS1/2 are not involved in Mtb induced expression of CXCL1 and CXCL2. (**a–c**) Macrophages were transfected with LATS1/2 KD constructs for 36 h followed by infection with Mtb H37Ra for 12 h to analyze the transcript levels of the indicated chemokines by qRT- PCR (**a**) or to detect the levels of CXCL1 (**b**) and CXCL2 (**c**) by ELISA. (**d** and **e**) LATS1 was knocked down in RAW264.7 macrophages by *Lats1* siRNA followed by infection with Mtb H37Ra for 12 h to check the levels of CXCL1 (**d**) or CXCL2 (**e**) in the culture supernatant. (**f**) Validation of LATS1 knock down in RAW264.7 macrophages. All the data represent mean ± SE (N = 3), *p < 0.05, ***p < 0.0001 (One way Anova). KD, kinase dead; siRNA, small interfering RNA; Med, medium; pg, picogram; h, hour; ns, not significant; NT, non-targeting; T, total.

The signaling cohort associated with MST1/2 include LATS1^40^. Therefore, to explore the role of the immediate target of MST1/2 i.e. LATS1 in inducing the expression of chemokines, kinase dead form of LATS1/2 was over expressed in macrophages followed by infection with Mtb. Interestingly, blockade of LATS1/2 function did not affect the expression of any of the infection-induced chemokines (Fig. 5a). Further, culture supernatants of Mtb-infected macrophages over expressing kinase dead forms of LATS1/2 showed no impairment in the production of MST1/2-responsive CXCL1 and CXCL2 (Fig. 5b,c), clearly showing that catalytic activity of LATS1/2 are not essential for MST1/2-responsive CXCL1 and CXCL2 production during mycobacterial infection of macrophages. Consistent with these observations, LATS1 knock down mediated by *Lats1* siRNA in RAW264.7 macrophages showed no impairment in Mtb- induced production of CXCL1 and CXCL2 (Fig. 5d,e). The efficiency of LATS1 knock down is shown in (Fig. 5f). These results strongly advocate the involvement of a non-canonical effector for mediating the expression of MST1/2-responsive chemokines during mycobacterial infection.

### MST1/2 regulates IRF3 in response to mycobacterial infection of macrophages

**Figure 6:**
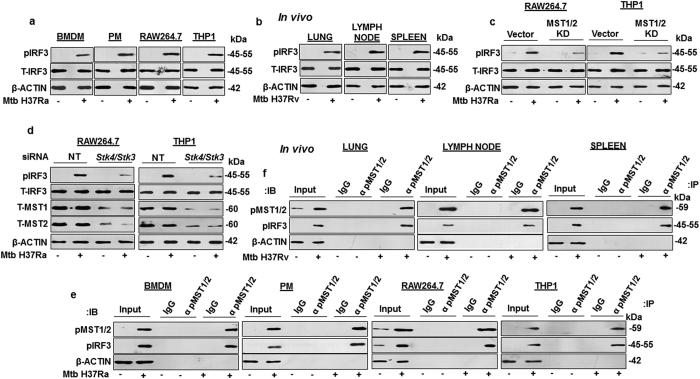
MST1/2 activate IRF3 during mycobacterial infection of macrophages. (**a**) Immunoblotting was performed to assess IRF3 activation in BMDM, PM, RAW264.7 and THP1 macrophages infected with Mtb H37Ra for 1 h. (**b**) IRF3 activation was assessed by immunoblotting in cells isolated from spleen, lymph node and lung of mice infected with Mtb H37Rv. (**c**) RAW264.7 and THP1 macrophages were transfected with MST1/2 KD constructs followed by infection with Mtb H37Ra for 1 h and IRF3 activation was assessed by immunoblotting. (**d**) MST1/2 were knocked down in RAW264.7 and THP1 macrophages by *Stk4*/*Stk3* siRNA followed by infection with Mtb H37Ra for 1 h to assess the IRF3 activation by immunoblotting. (**e**) Immuno-pulldown experiment was performed in BMDM, PM, RAW264.7 and THP1 macrophages to analyze the Mtb-driven interaction of pMST1/2 with pIRF3. (**f**) Immuno-pulldown experiment was performed to assess the interaction of pMST1/2 with pIRF3 in lung, spleen and lymph node of mice infected with Mtb H37Rv. Blots are representatives of three independent experiments. p, phospho; T, total; BMDM, bone marrow derived macrophage; PM, peritoneal macrophage; NT, non-targeting; siRNA, small interfering RNA; h, hour; IB, immunoblotting; IP, immuno-pulldown; IgG, immunoglobulin G; α, anti.

IRF3 is a transcription factor known for its role in defending the host against viral infections by stimulating the production of interferons^41^,^42^,^43^. It has been known to be responsive to LPS-induced TLR4 signaling^44^ and has been implicated in host responses during mycobacterial infection^45^,^46^ and also in the modulation of chemokines during viral and certain bacterial infections^45^,^47^,^48^. Interestingly, a recent report showed that MST1/2 is capable of regulating IRF3 in response to viral infection^49^. However, the regulation of IRF3 activity by MST1/2 during mycobacterial infection of macrophages is not known. This premise led us to investigate the role for MST1/2 in regulating IRF3 activity in mycobacteria-infected macrophages. To this end the phosphorylation status of IRF3 was analysed in Mtb-infected BMDM, PM, RAW264.7 and THP1 macrophages (Fig. 6a) and in lung, lymph node and spleen (Fig. 6b) of Mtb aerosolized mice; which clearly showed the activation of IRF3 without changing its total cellular pool. In order to assess the role for MST1/2 in regulating IRF3 activity, RAW264.7 and THP1 macrophages transiently transfected with MST1/2 kinase dead construct, were infected with Mtb (Fig. 6c). Interference with MST1/2 function clearly showed reduction in the Mtb-induced IRF3 activation. These results clearly suggest that catalytic activities of MST1/2 are essential for IRF3 activation during mycobacterial infection. Further, knock down of MST1/2 by *Stk4*/*Stk3* siRNA in RAW264.7 and THP1 macrophages also led to a significant loss in infection-driven IRF3 activation (Fig. 6d). Next we assessed the mechanism by which MST1/2 regulate the activity of IRF3. To this end immuno-pulldown experiments clearly showed the interaction of phospho MST1/2 with phospho IRF3 in Mtb-infected BMDM, PM, RAW264.7, THP1 macrophages (Fig. 6e) and in lung, lymph node and spleen of Mtb aerosolized mice (Fig. 6f). Altogether, these results clearly suggest infection-induced MST1/2 interaction-dependent activation of IRF3.

### IRF3 effectuates pathogen-specific-MST1/2-induced CXCL1 and CXCL2 production

**Figure 7:**
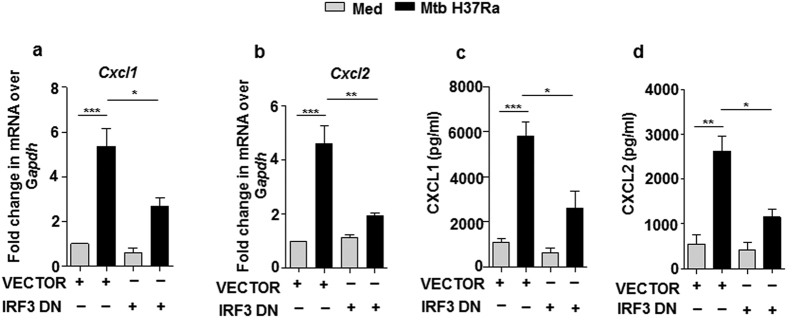
Mtb activated IRF3 drives MST1/2-regulated production of CXCL1 and CXCL2. (**a–d**) RAW264.7 macrophages were transfected with IRF3 DN construct followed by infection with Mtb H37Ra for 12 and expression of *Cxcl1* (**a**) or *Cxcl2* (**b**) at mRNA level and secretion of CXCL1 (**c**) and CXCL2 (**d**) in supernatant was analyzed by qRT-PCR and ELISA respectively. All the data represent means ± SE (N = 3), *p < 0.05, **p < 0.005, ***p < 0.0001 (One way Anova). Med, medium; pg, picogram; DN, dominant negative.

With the premise that IRF3 regulates the expression of several chemokines during infection with viruses and certain bacteria^50^,^51^ led us to explore the role of IRF3 in mycobacteria-induced production of chemokines. To this end, dominant negative form of IRF3 (lacking DNA binding domain) was over expressed in macrophages, followed by infection with mycobacteria (Fig. 7a–d); which rendered Mtb sufficiently incapable of inducing the expression of the concerned chemokines. These results explicitly bespeak the role for MST1/2-regulated IRF3 in the production of CXCL1 and CXCL2 during mycobacterial pathogenesis.

### CXCL1 and CXCL2 activate host innate immune effectors

**Figure 8:**
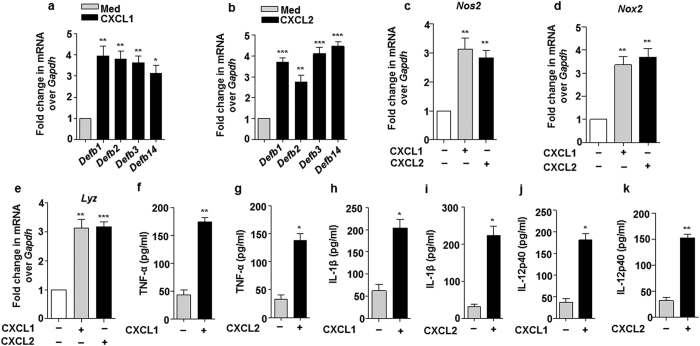
CXCL1 and CXCL2 induce the production of anti-microbial molecules and inflammatory cytokines. (**a–e**) Transcript levels of *Defb1*, *Defb2*, *Defb3*, *Defb14* (**a**-**b**), *Nos2* (**c**) and *Nox2* (**d**) and *Lyz* (**e**) were analyzed by qRT-PCR in macrophages which were treated for 12 h with either recombinant CXCL1 or CXCL2 (4 ng/ml each). (**f**–**k**) Secretory levels of TNF-α (**f** and **g**) or IL-1β (**h** and **i**) or IL-12p40 (**j** and **k**) were analyzed by ELISA in the cell free culture supernatants obtained from macrophages treated with either 4 ng/ml of CXCL1 or CXCL2 each for 12 h. All the data represent means ± SE (N = 3), *p < 0.05, **p < 0.005, ***p < 0.0001 (One way Anova or t test). Med, medium; pg, picogram.

**Figure 9:**
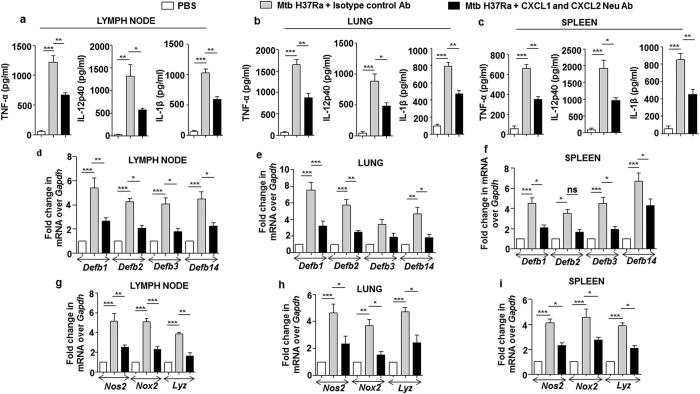
CXCL1 and CXCL2 are positive modulators of mycobacteria induced production of inflammatory molecules. (**a–c**) CXCL1 and CXCL2 were neutralized by using 20 μg of CXCL1 and CXCL2 neutralizing antibody, respectively *in vivo* to assess the status of Mtb H37Ra induced production of pro-inflammatory cytokines TNF-α, IL-12p40 and IL-1β in lymph node (**a**), lung (**b**) and spleen (**c**) of mice by ELISA. (**d**–**i**) The role of CXCL1 and CXCL2 in regulating the Mtb H37Ra induced production of inflammatory mediators such as defensins in lymph node (**d**), lung (**e**), spleen (**f**) and *Nox2*, *Nos2*, *Lyz* in lymph node (**g**), lung (**h**) and spleen (**i**) were also assessed by qRT-PCR. All the data represent means ± SE (N = 3), *p < 0.05, **p < 0.005, ***p < 0.0001 (One way Anova or t test). Neu, neutralizing; Ab, antibody; pg, picogram; PBS, phosphate buffered saline.

Chemokines are widely known for their ability to direct the recruitment of immune cells towards the site of immunity breach. However, amassing evidences suggest that they may also arm such immune cells with anti-microbial capacities to further enhance their efficacy to contain the immune insult^52^,^53^. In this regard, it was intriguing to explore the capacity of mycobacteria-induced CXCL1 and CXCL2 in armoring neighboring macrophages with innate immune defenses like anti-microbial peptides (AMPs) such as beta-defensins (Defb) and inflammatory mediators including TNF-α, IL-1β, IL-12p40, iNOS (inducible nitric oxide synthase), NOX (NADPH oxidase) and LYZ (Lysozyme). Interestingly, treatment of macrophages with recombinant CXCL1 or CXCL2 induced the expression of *Defb* (Fig. 8a,b), *Nos2* (Fig. 8c), *Nox2* (Fig. 8d), *Lyz* (Fig. 8e) and proinflammatory cytokines (Fig. 8f–k). Having assessed the role for CXCL1 and CXCL2 *in vitro*, in regulating the host innate immune responses; the *in vivo* requirements of these chemokines in modulating the concerned host innate immune responses during mycobacterial infection was envisaged. To this end, Mtb-induced secretory CXCL1 and CXCL2 were neutralized by utilizing respective neutralizing antibodies. Although, neutralization of CXCL1 and CXCL2 did not completely abolish the Mtb-induced production of innate immune regulators; however, a significant reduction in the production of proinflammatory cytokines (Fig. 9a–c), *Defb* (Fig 9d–f), and *Nos2*, *Nox2*, *Lyz* (Fig. 9g–i) was observed in lymph node, lung and spleen of Mtb-infected mice neutralized for CXCL1 and CXCL2. These results clearly demonstrate that CXCL1 and CXCL2 are one of the important regulators of host innate immune responses during mycobacterial infection.

### Dependence of CXCL1 and CXCL2 mediated generation of host innate immune effectors on MST1/2

**Figure 10:**
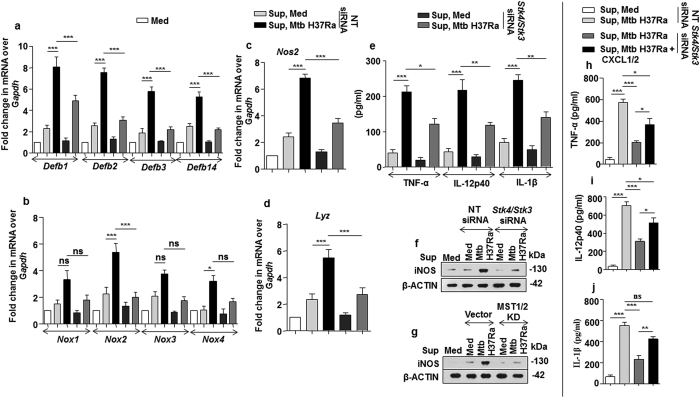
Regulation of innate immune responses by CXCL1 and CXCL2 are dependent on Mtb induced MST1/2 activity. (**a–g**) Fresh macrophages were treated with equal volumes of cell culture supernatants derived from MST1/2 knocked down macrophages which were infected for 12 h with Mtb or left uninfected to study gene expression of *Defb1*, *Defb2*, *Defb3*, *Defb14* (**a**), *Nox1*, *Nox2*, *Nox3*, *Nox4* (**b**), *Nos2* (**c**) and *Lyz* (**d**) by qRT-PCR or ELISA was performed to detect the levels of secreted TNF-α, IL-12p40 and IL-1β (**e**) in the culture supernatants and protein expression of iNOS (**f,g**) was analyzed by immunoblotting. (**h**–**j**) Supernatant obtained from MST1/2 knocked down Mtb infected macrophages were treated with recombinant CXCL1 and CXCL2 or left untreated. These supernatants were used to treat fresh macrophages and ELISA was performed to detect TNF-α (**h**), IL-12p40 (**i**) and IL-1 β (**j**). Blots are representatives of three independent experiments. All the data represent means ± SE (N = 3), *p < 0.05, **p < 0.005, ***p < 0.0001 (One way Anova). Med, medium; pg, picogram; sup, supernatant; KD, kinase dead; siRNA, small interfering RNA; NT, non-targeting; ns, not significant.

Since, CXCL1 and CXCL2 production were regulated by MST1/2; the role of Mtb-activated MST1/2 in regulating the CXCL1- and CXCL2- induced innate immune functions of macrophages was assessed. We found that the capacity of CXCL1 and CXCL2 to induce the concerned immune effectors was dependent on MST1/2 activity; as macrophages treated with culture supernatants derived from Mtb-infected MST1/2-knocked-down cells showed significant reduction in the production of *Defb* (Fig. 10a), *Nox* (Fig. 10b), *Nos2* (Fig. 10c,f,g), *Lyz* (Fig. 10d), and pro-inflammatory molecules (Fig. 10e). Further, to specifically validate the requirement of CXCL1 and CXCL2 in inducing innate immune regulators, culture supernatants derived from Mtb-infected MST1/2 knocked down macrophages were exogenously treated with recombinant CXCL1 and CXCL2. Naïve macrophages treated with this supernatant could significantly rescue the level of pro-inflammatory cytokines i.e. TNF-α (Fig. 10h), IL-12p40 (Fig. 10i) and IL-1β (Fig. 10j). Taken together, our observations reveal that CXCL1 and CXCL2 secreted from Mtb-infected macrophages in an MST1/2-dependent manner, initiate paracrine signaling in neighboring macrophages to contribute towards the production of innate immune effectors like defensins, NOX, iNOS and pro-inflammatory cytokines as part of host defense upon mycobacterial infection (Fig. S27).

## Discussion

The control of pathogen dissemination assumes prime consideration with the shortfall of immune system to effect absolute sterilization of mycobacterial infection. The formation of granuloma is a move towards this goal, which is achieved by a coordinated recruitment of immune cells^52^. Chemokines like MCP1, RANTES, MIP1α and CXCL1 are known potent chemo-attractants for T-cells, monocytes; and neutrophils, respectively^54^. In addition to their canonical function, some chemokines like CXCL8 can also enhance bactericidal activity through non-oxidative mechanisms. In line, CXCL8 gene polymorphisms have been associated with susceptibility to TB^55^. Chemokines are therefore, now being studied for their potential use as adjuvants for anti-mycobacterial therapy and the regulatory pathways for these may also be conceivably exploited in this regard^56^.

Accumulating evidence suggests the role for MAPKs, PI3K, NF-κB pathways and cytokines like TNF-α and IFN-γ to modulate chemokine production. Our investigation projects the activation of Hippo signaling pathway upon mycobacterial infection condition *in vitro* as well as *in vivo*. Upon infection, Mtb interacts with several pattern recognition receptors (PRRs), of which engagement with TLR2 serves majorly towards Mtb-mediated immune modulation. In this premise, we found that the activation of the pioneer molecule of Hippo signaling pathway, MST1/2, occurs through TLR2 as mycobacterial infection of cells obtained from *tlr2* knockout mice failed to activate MST1/2 and its downstream targets. Further delineation unveiled TLR2 adaptors, IRAK1 and IRAK4, to directly interact with MST1/2 to bring about its activation post Mtb infection.

This study identifies Hippo signaling to be a novel regulatory circuit in the production of two of the Mtb-induced chemokines, CXCL1 and CXCL2. These chemokines are known to recruit neutrophils to site of infection^57^,^58^, which is an important early step in controlling infections. Interestingly, it was found that although CXCL1 and CXCL2 were MST1/2-dependent, their expression stood exclusive of LATS1/2; indicating the process to be arbitrated through some non-canonical effectors. Indeed, MST kinases are known to regulate various transcription factors, including IRF3^49^. IRF3 is well known for its role in defending the host against viral infection and certain bacterial infections like that with *Pseudomonas aeruginosa*^48^,^59^. Also, as introduced, it is known to be activated during mycobacterial infection to regulate various host responses. Further, the premise that IRF3 may regulate the expression of several chemokines led us to explore its role in Mtb-induced MST1/2-mediated regulation of CXCL1 and CXCL2 production. The present study highlights IRF3 to be a downstream target of MST1/2, effectuating the MST1/2-dependent production of chemokines during mycobacterial infection. However, the prospective mechanism by which IRF3 regulates chemokine production remains elusive^60^.

It was interestingly striking to find that MST1/2-dependent secretion of CXCL1 and CXCL2 from Mtb-infected macrophages initiated paracrine signaling events leading to the production of innate immune effectors like beta-defensins, NOXs, iNOS and pro-inflammatory cytokines from neighboring receptive macrophages. These molecules may provide further prospect of elimination of the pathogen. Therefore, signaling cross-talks and orchestrations within and among several infected and/or uninfected immune cells leading to the concerted response towards mycobacterial infection must be conceived for advancement of anti-TB measures.

Taken together, we lucidly unearth the activation of Hippo signaling during mycobacterial infection and identify TLR2-IRAK1/4-MST1/2 axis in governing the production of CXCL1 and CXCL2 through a non-canonical MST1/2 effector, IRF3. These chemokines further guide the by-stander macrophages to produce defensive cytokines and AMPs; alongside their canonical function as chemo-attractants. The unveiling of such novel regulatory networks may provide potential platforms for the development of host-directed therapeutic and vaccination strategies for TB.

## Methods

### Mice and cell lines

Primary macrophages were isolated from peritoneal exudates of 4 to 6 weeks old BALB/c, C57BL/6 or *Tlr2*-knockout mice after intra-peritoneal stimulation with 1 ml of 8% Brewers thioglycollate. Four days post-immunization, mice were sacrificed to obtain peritoneal exudates by flushing the peritoneal cavity with ice-cold PBS. Isolated cells were cultured in DMEM (Gibco, USA) containing 10% FBS (Gibco, USA) for 6 to 8 h. Adherent cells were used as peritoneal macrophages (PMs). Bone marrow derived macrophages (BMDMs) were isolated from femurs and tibias obtained from WT mice and cultured in DMEM medium containing 30% L-929 fibroblast conditioned medium. The purity of these cells were confirmed by F4/80 staining using fluorescence activated cell sorter (FACS) and was found to be >95%. All studies involving mice were carried out with the approval from the Institutional Ethics Committee for animal experimentation, Indian Institute of Science as well as from the Institutional Biosafety Committee, Indian Institute of Science. *In vitro* studies were carried out with murine RAW 264.7 macrophage like cells and THP1 macrophages, obtained from National Center for Cell Sciences, Pune, India.

### Ethics statement

All studies involving mice and virulent mycobacteria were carried out after the approval from the Institutional Ethics Committee for animal experimentation as well as from Institutional Biosafety Committee. The animal care and use protocol adhered were approved by national guidelines of the Committee for the purpose of Control and Supervision of Experiments and Animals (CPCSEA), Government of India.

### *In vitro* and *in vivo* infections

*Mycobacterium tuberculosis* (Mtb) H37Rv and Mtb H37Ra were grown to mid log phase and used at multiplicity of infection (MOI) of 10 for all the *in vitro* experiments. For *in vivo* infections, WT or *Tlr2* knockout mice were aerosolized with 500 CFUs of Mtb H37Rv and maintained in securely commissioned BSL3 facility for 44 days, followed by their euthanisation to isolate lung, lymph node and spleen. Cells obtained from these organs were used for the experiments as indicated. Control mice received PBS.

### Reagents and antibodies

General laboratory chemicals were procured from Sigma-Aldrich (USA) or Merck (Germany). MST1 and MST2 kinase dead and LATS2 kinase dead constructs were procured from Addgene (USA). Non-targeting small interfering RNA (siRNA) (D-001210-01-20), *Stk4*/*Mst1* (M-059385-01-0005), *Stk3*/*Mst2* (M-040440-01-0005) and *MyD88* (M-063057-00-0005), *Irak1* (M-040116-01-0005), *Irak4* (M-061264-01-0005) and *Lats1* (M-063467-01-0005) siRNAs were obtained from Dharmacon as siGENOME SMART-pool reagents. Anti β-ACTIN (A3854), anti iNOS (N7782) and anti phospho IRAK1 (SAB4504246) were purchased from Sigma-Aldrich (USA). Anti phospho MST1 (Thr183)/MST2 (Thr180) (3681), MST1 (14946), MST2 (3952), anti phospho LATS1 (Thr1079) (8654), anti LATS1 (3477), anti phospho IRF3 (Ser396) (4947), anti IRF3 (4302) anti IRAK1 (4504), anti phospho IRAK4 (7652), anti IRAK4 (4363), anti phospho SRC (Tyr416) (2101) and anti MYD88 (4283) antibodies were purchased from Cell Signaling Technology (USA). CXCL1 neutralizing (AF-453-NA) and CXCL2 neutralizing (AF-452-NA) antibodies were purchased from R & D Systems Inc. (USA). Goat IgG (02-6202) isotype control antibody was purchased from Thermo Fisher Scientific (USA). Horseradish peroxidase (HRP) conjugated anti- rabbit IgG (111-035-045) and light chain specific anti-rabbit IgG (211-032-171) were purchased from Jackson ImmunoResearch (USA). PE-conjugated F4/80 antibody was purchased from Tonbo Biosciences.

### Transient transfection studies

Murine RAW264.7 and THP1 macrophages were transfected with 100 nM non-targeting siRNA or target specific siRNAs; or 4 μg vector controls or indicated constructs by using low molecular weight polyethylenimine (Sigma-Aldrich, USA). Transient transfection efficiency of RAW264.7 was found to be 70–80% in all the experiments as determined by counting the number of siGLO lamin A/C positive cells in a microscopic field using fluorescence microscope. In all the cases, post 36 h of transfection, cells were treated or infected, as indicated, for the required time durations.

### Treatment with pharmacological reagents, recombinant proteins and culture supernatants

#### Pharmacological reagents

Macrophages were treated *in vitro* with 5 μM IRAK1/4 inhibitor (509093-47-4) (Merck, Germany) for 1 h followed by infection. 0.1% DMSO (Sigma-Aldrich, USA) was used as vehicle control. A tested concentration of inhibitor was identified by a careful titration experiment assessing the viability of macrophages using MTT [3-(4, 5-dimethylthiazol-2yl)-2, 5-diphenyltetrazolium bromide] assay prior to utilization for experiments. For *in vivo* studies, mice were administered with IRAK1/4 inhibitor intra-venously at a dose of 5 mg/kg for 12 h, followed by intra-venous infection with 10^7^ CFU of Mtb H37Ra for 3 days. The treated mice were euthanized to isolate spleen, lungs and lymph node for the requisite experiments. DMSO was used as vehicle control. Each *in vivo* experiment was performed independently in 5 mice.

#### Recombinant proteins

Macrophages were treated with either 4 ng/ml CXCL1 (250-11) or CXCL2 (250-15) (Peprotech, USA) for the indicated time points and requisitely harvested for qRT-PCR, ELISA and immunoblotting experiments.

#### Culture supernatants

Equal amount of cell free supernatants obtained from infected or uninfected MST1/2 knocked down macrophages were used to treat fresh macrophages. Else, supernatant obtained from MST1/2 knocked down macrophages infected with Mtb were treated exogenously with 4 ng/ml of CXCL1 and CXCL2 each and utilized thereafter for stimulating fresh macrophages and qRT-PCR and ELISA was performed.

### CXCL1 and CXCL2 neutralization

Mice were injected intra-venously with 20 μg of goat anti-mouse CXCL1 and CXCL2 neutralizing antibody 1 h prior to intra-venous infection with 10^7^ CFU of Mtb H37Ra. After 12 h mice were sacrificed to isolate lung, spleen and lymph node and processed for the indicated experiments. 20 μg isotypic IgG antibody was used as vehicle control. Control mice were injected with PBS.

### RNA isolation and quantitative real time reverse transcription polymerase chain reaction (qRT-PCR)

Total RNA was isolated from macrophages by TRI reagent (Sigma-Aldrich, USA). 2 μg of RNA was converted into complementary DNA (cDNA) by using first strand cDNA synthesis kit (Bioline, UK). Quantitative real time RT-PCR (qRT-PCR) was performed using a SYBR green PCR mix (Kapa Biosystems, USA) for quantification of target gene expression. Expression of *Gapdh* was used as the normalization control for qRT-PCR analysis. The primer sequences used for the current study are; *Ccl2* Fwd 5′-taaaaacctggatcggaaccaaa-3′, *Ccl2* Rev 5′-gcattagcttcagatttacgggt-3′, *Ccl1* Fwd 5′-tgccgtgtggatacaggatg-3′, *Ccl1* Rev 5′-gttgaggcgcagctttctcta-3′, *Cxcl1* Fwd 5′-ctgggattcacctcaagaacatc-3′, *Cxcl1* Rev 5′-cagggtcaaggcaagcctc-3′, *Cxcl2* Fwd 5′-ccaaccaccaggctacagg-3′, *Cxcl2* Rev 5′-gcgtcacactcaagctctg-3′, *Ccl3* Fwd 5′-tgtaccatgacactctgcaac-3′, *Ccl3* Rev 5′-caacgatgaattggcgtggaa-3′, *Cxcl15* Fwd 5′-tcgagaccatttactgcaacag-3′, *Cxcl15* Rev 5′-cattgccggtggaa attcctt-3′, *Ccl8* Fwd 5′-tctacgcagtgcttctttgcc-3′, *Ccl8* Rev 5′-aagggggatcttcagctttagta-3′, *Ccl17* Fwd 5′-taccatgaggtcacttcagatgc-3′, *Ccl17* Rev 5′-gcactctcggcctacattgg-3′, *Ccl12* Fwd 5′-atttccacacttctatgcctcct-3′, *Ccl12* Rev 5′-atccagtatggtcctgaagatca-3′, *Ccl7* Fwd 5′-gctgctttcagcatccaagtg-3′, *Ccl7* Rev 5′-ccagggacaccgactactg-3′ *Cxcl3* Fwd 5′-gatctcaccacagcccttcg-3′, *Cxcl3* Rev 5′-ctgtagcctggtggttggtg-3′, *Defb1* Fwd 5′-aggtgttggcattctcacaag-3′, *Defb1* Rev 5′-gcttatctggtttacaggttccc-3′, *Defb2* Fwd 5′-gctgatatgctgcctccttt-3′, *Defb2* Rev 5′-gaggacaaatggctctgaca-3′, *Defb3* Fwd 5′-ttcctcaaatgctgcaagag-3′, *Defb3* Rev 5′-tgctagggagcacttgtttg-3′, *Defb14* Fwd 5′-tcatcttgttcttggtgcct-3′, *Defb14* Rev 5′-tcttcctttccaaggcagtt-3′, *Nox1* Fwd 5′-ggttggggctgaacatttttc-3′, *Nox1* Rev 5′-tcgacacacaggaatcaggat-3′, *Nox2* Fwd 5′-tgaatgccagagtcgggattt-3′, *Nox2* Rev 5′-cgagtcacggccacataca-3′, *Nox3* Fwd 5′-tggcagtaaacgcctatctgt-3′, *Nox3* Rev 5′-cggaacccagaataactcgtgta-3′, *Nox4* Fwd 5′-ccttttacctatgtgccggac-3′, *Nox4* Rev 5′-catgtgatgtgtagagtcttgct-3′, *Lyz* Fwd 5′-atggaatggctggctactatgg-3′, *Lyz* Rev 5′-accagtatcggctattgatctga-3′, *Nos2* Fwd 5′-ggagtgacggcaaacatgact-3′, *Nos2* Rev 5′-tcgatgcacaactgggtgaac-3′, *Gapdh* Fwd 5′-gagccaaacgggtcatcatct-3′, *Gapdh* Rev 5′-gaggggccatccacagtctt-3′.

### ELISA

Sandwich ELISA was performed in 96 well microtiter plates (Nunc, Denmark) using cell free supernatants of respectively treated samples. CXCL1 (900-K127) and CXCL2 (900-K152) ELISA kits were purchased from Peprotech (USA); and TNF-α (555268), IL-1β (559603) and IL-12p40 (555165) were obtained from BD Biosciences (USA). ELISA was performed according to the manufacturer’s instructions.

### Flow Cytometric Analysis

10^6^ mouse peritoneal macrophages were taken for per sample and suspended in blocking solution. 1 μg of Fc receptor blocking antibody was added to each sample and incubated for 15 minute on ice. Next, 1 μg of anti- F4/80 antibody conjugated with PE or isotype control antibody conjugated with PE was added in each sample and incubated for 30 min at 4 °C in dark. Post incubation, samples were centrifuged at 1500 RPM for 5 min at 4 °C with washing and suspended in ice-cold PBS to perform flow cytometric analysis.

### Immunoblotting

Treated or untreated or infected or uninfected macrophages were lysed in radioimmunoprecipitation assay (RIPA) buffer containing 50 mM Tris-HCl (pH 7.4), 1% NP-40, 0.25% sodium deoxycholate, 150 mM NaCl, 1 mM EDTA, 1 mM PMSF, 1 μg/ml each of aprotinin, leupeptin, and pepstatin, 1 mM Na_3_VO_4_, and 1 mM NaF. An equal amount of protein from each cell lysates were resolved on a 12% SDS-PAGE and transferred onto PVDF membrane (Millipore, USA) by the semidry transfer (Bio-Rad, USA) method. Nonspecific binding was blocked with 5% nonfat dry milk powder in TBST (20 mM Tris- HCl [pH 7.4], 137 mM NaCl, 0.1% Tween 20) for 60 min. The blots were incubated overnight at 4 °C with primary antibody, followed by probing with goat anti-rabbit HRP-conjugated secondary antibody in 5% non-fat milk for 2 h. After washing in TBST, the immunoblots were developed with an ECL detection system (PerkinElmer, USA) as per the manufacturer’s instruction.

### Immunoprecipitation assay

Immunoprecipitation assays were carried out following a modified version of the protocol provided by Millipore, USA. In brief, macrophages were gently suspended and lysed in ice-cold RIPA buffer. The cell lysates obtained were incubated with IgG or anti phospho IRAK1 or anti phospho IRAK4 or anti phospho MST1/2 antibodies for 2 h at 4 °C. The immune complexes were captured on protein A agarose beads (Bangalore Genei, India) at 4 °C for 4 h. The beads were separated, washed and boiled in Laemmli buffer for 10 min. These bead free samples were analyzed for respective target molecules by immunoblotting after separation by SDS-PAGE. Light chain specific secondary antibody was used for immunoblotting after immunoprecipitation.

### MTT Assay

10,000 cells were seeded to wells of 96 well microtiter plate. After 24 hours, when the monolayer formed the supernatant was flicked off and 100 μl of indicated supernatants were added to the cell in microtiter plate and kept for incubation at 37 °C in 5% CO_2_ incubator for 12 hours. After 12 hours, supernatant was flicked off and 50 μl of MTT dye was added to wells. The plates were gently shaken and incubated for 4 hours at 37 °C in 5% CO_2_ incubator. The supernatant was removed, 50 μl of DMSO was added, and the plates were gently shaken to solubilize the formed formazan. The absorbance was measured using a microplate reader at a wavelength of 595 nm and the percentage of cell survival was calculated.

### Statistical analysis

Significance for comparison between samples was determined by the Student *t* test distribution or one-way analysis of variance (ANOVA). The data in the graphs are expressed as the mean ± standard error (SE) and *P* values of <0.05 were defined as significant. Graphpad Prism (version 5.0) software (Graphpad Software, USA) was used for all the statistical analysis.

## Additional Information

**How to cite this article**: Boro, M. *et al*. *Mycobacterium tuberculosis*-triggered Hippo pathway orchestrates CXCL1/2 expression to modulate host immune responses. *Sci. Rep*. **6**, 37695; doi: 10.1038/srep37695 (2016).

**Publisher's note:** Springer Nature remains neutral with regard to jurisdictional claims in published maps and institutional affiliations.

## Supplementary Material

Supplementary Information
