# Supplementary material for: Mycobacterium tuberculosis-triggered Hippo pathway orchestrates CXCL1/2 expression to modulate host immune responses
Source: Sci Rep. 2016 Nov 24;6:37695. doi: 10.1038/srep37695 (PMC5121601; doi:10.1038/srep37695)
Supplement: Supplementary Information [file srep37695-s1.pdf]

Supplementary information for

Mycobacterium tuberculosis-triggered Hippo pathway orchestrates CXCL1/2 expression to modulate host immune responses

Monoranjan Boro[1], Vikas Singh[1] and Kithiganahalli Narayanaswamy Balaji[1*]

Figure S1

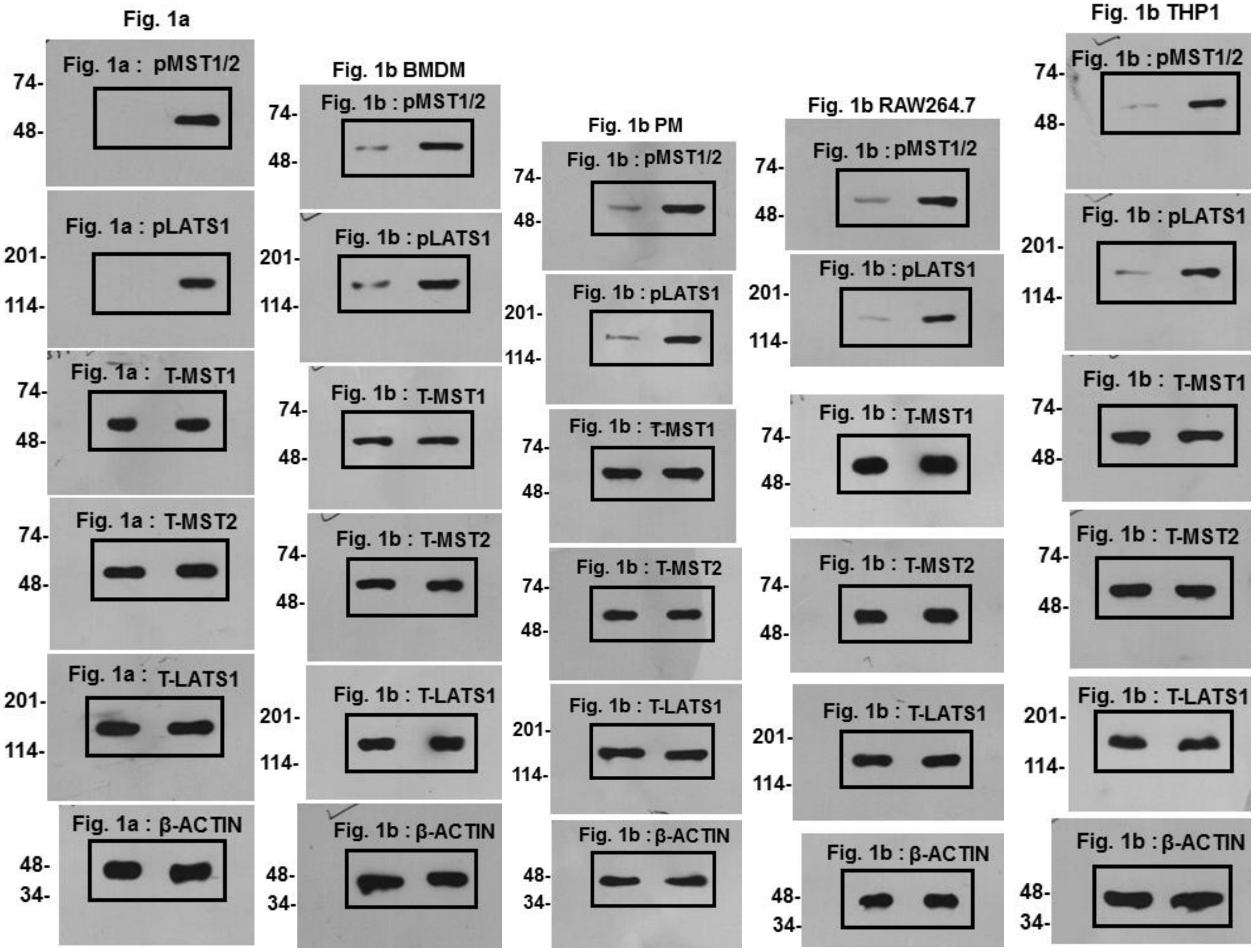

Supplementary Figure S1. Original western blots for images used in Figure 1a and 1b. Black boxes indicate the specific bands used in the main figure.

Figure S2

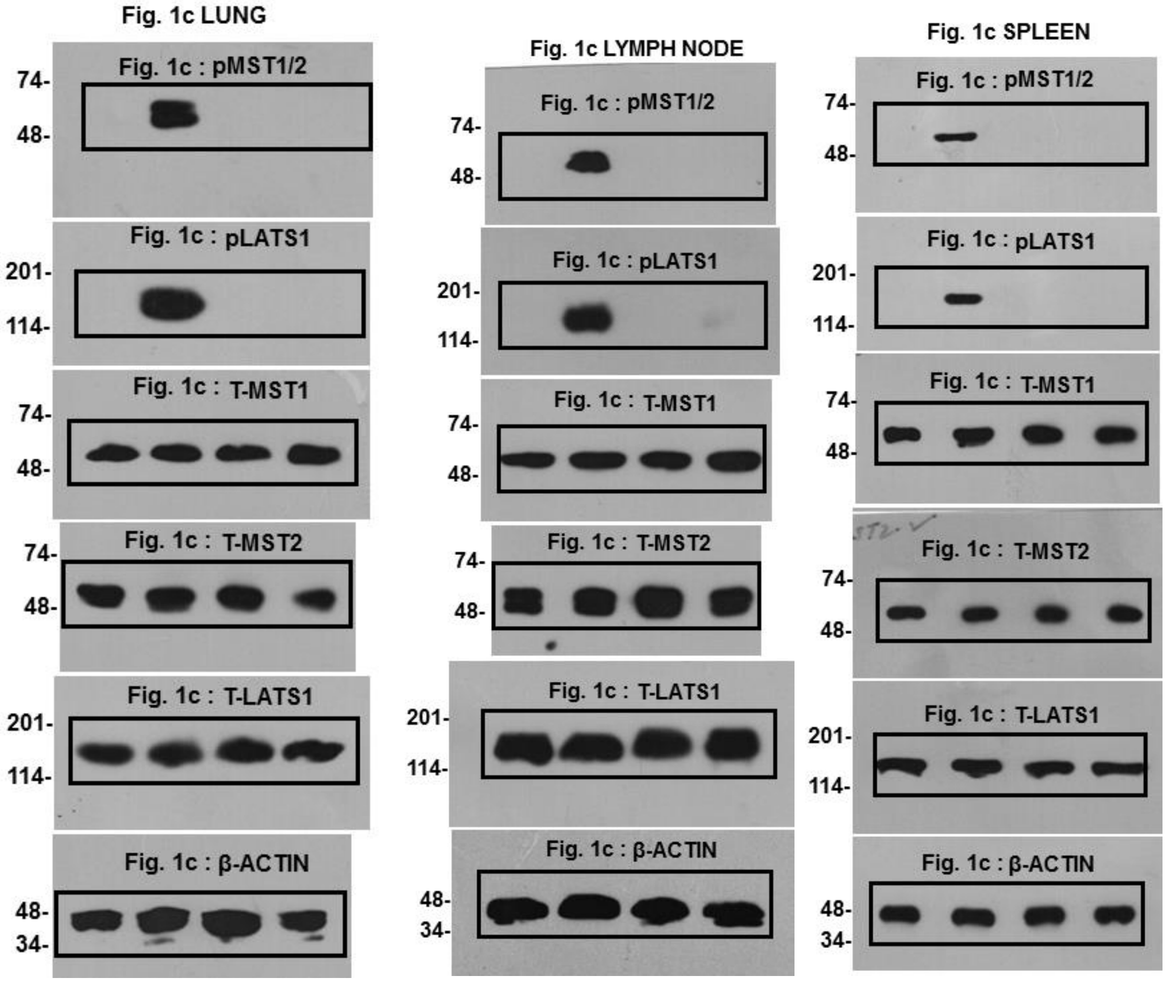

Supplementary Figure S2. Original western blots for images used in Figure 1c. Black boxes indicate the specific bands used in the main figure.

Figure S3

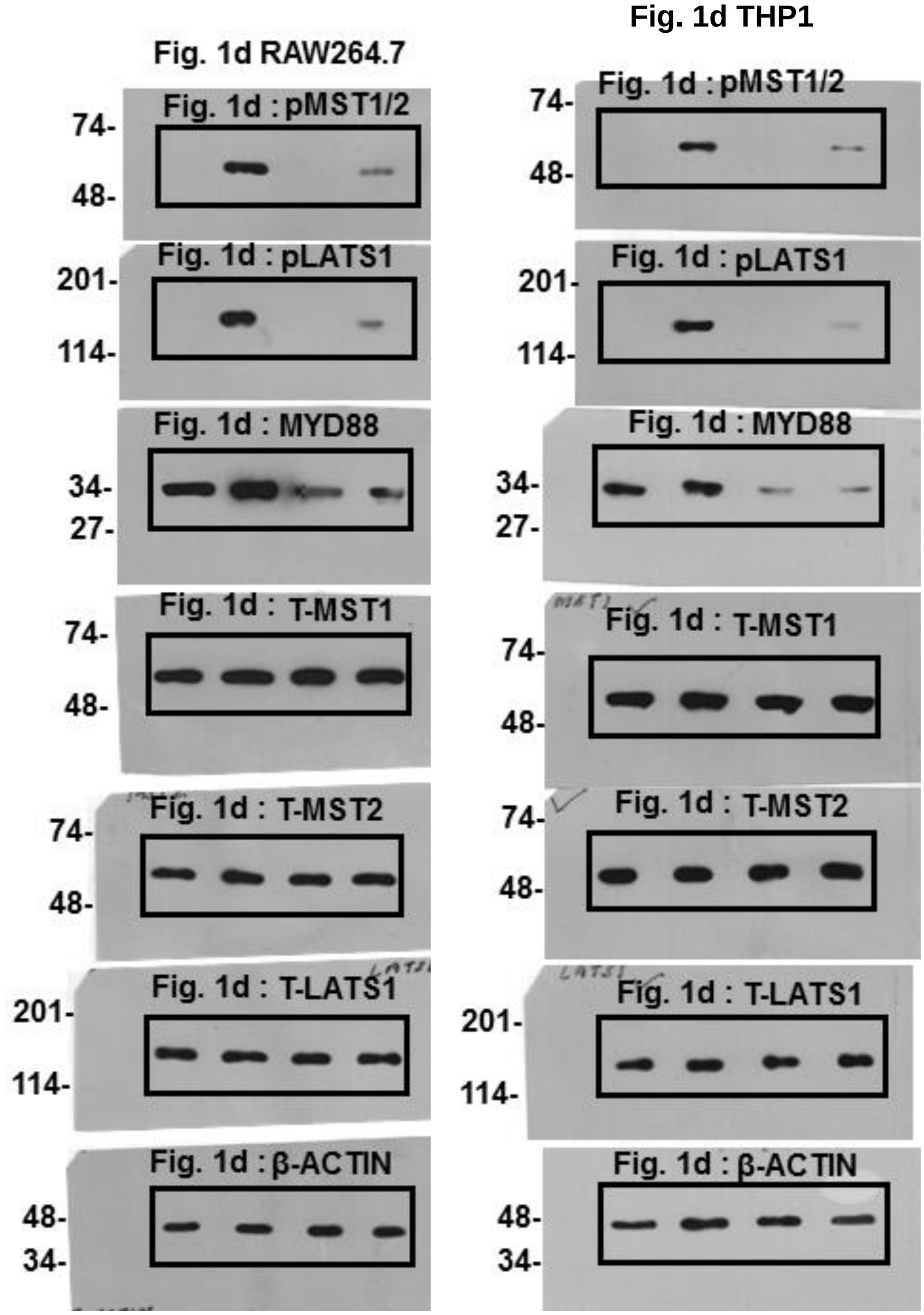

Supplementary Figure S3. Original western blots for images used in Figure 1d. Black boxes indicate the specific bands used in the main figure.

Figure S4

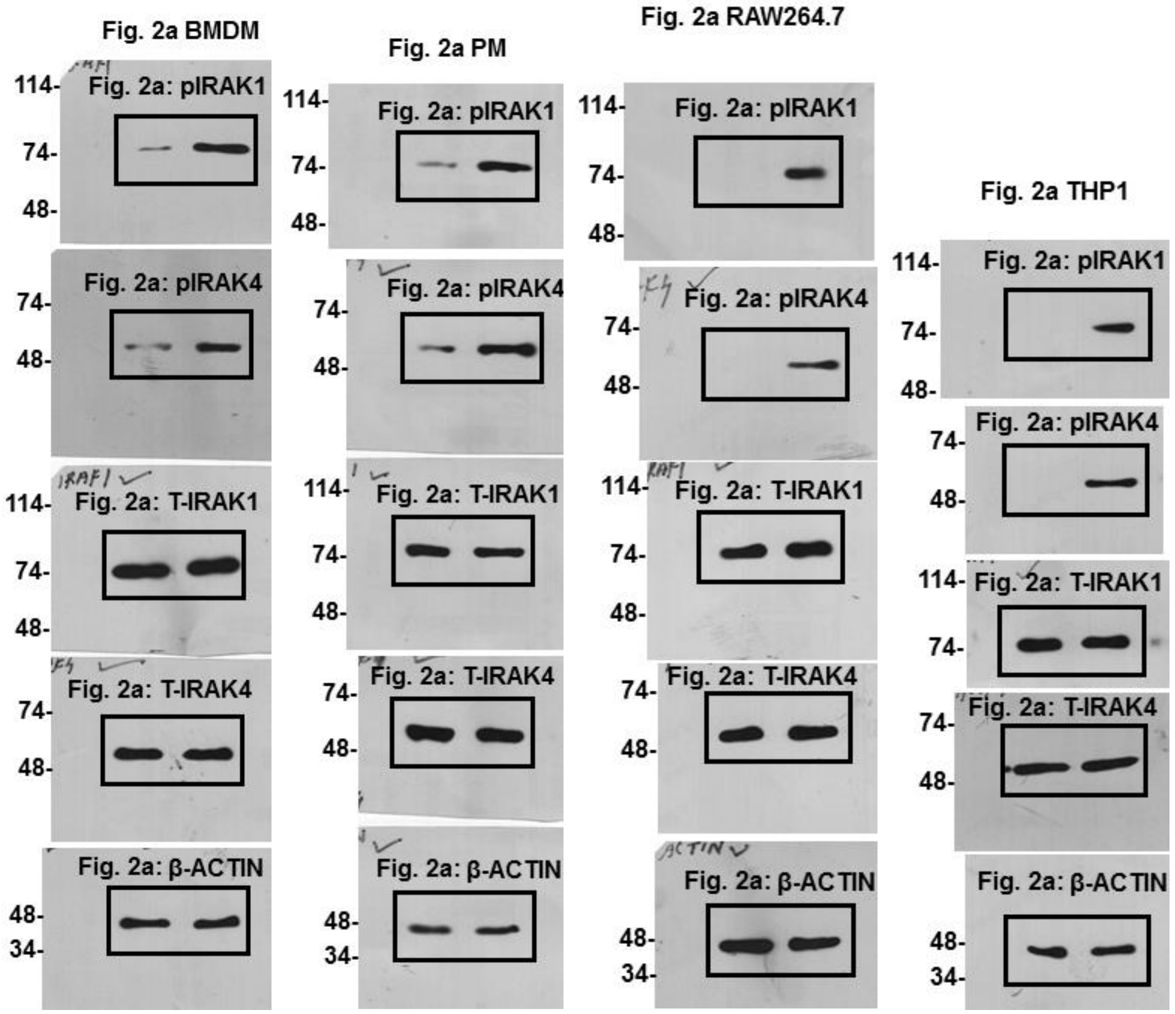

Supplementary Figure S4. Original western blots for images used in Figure 2a. Black boxes indicate the specific bands used in the main figure.

Figure S5

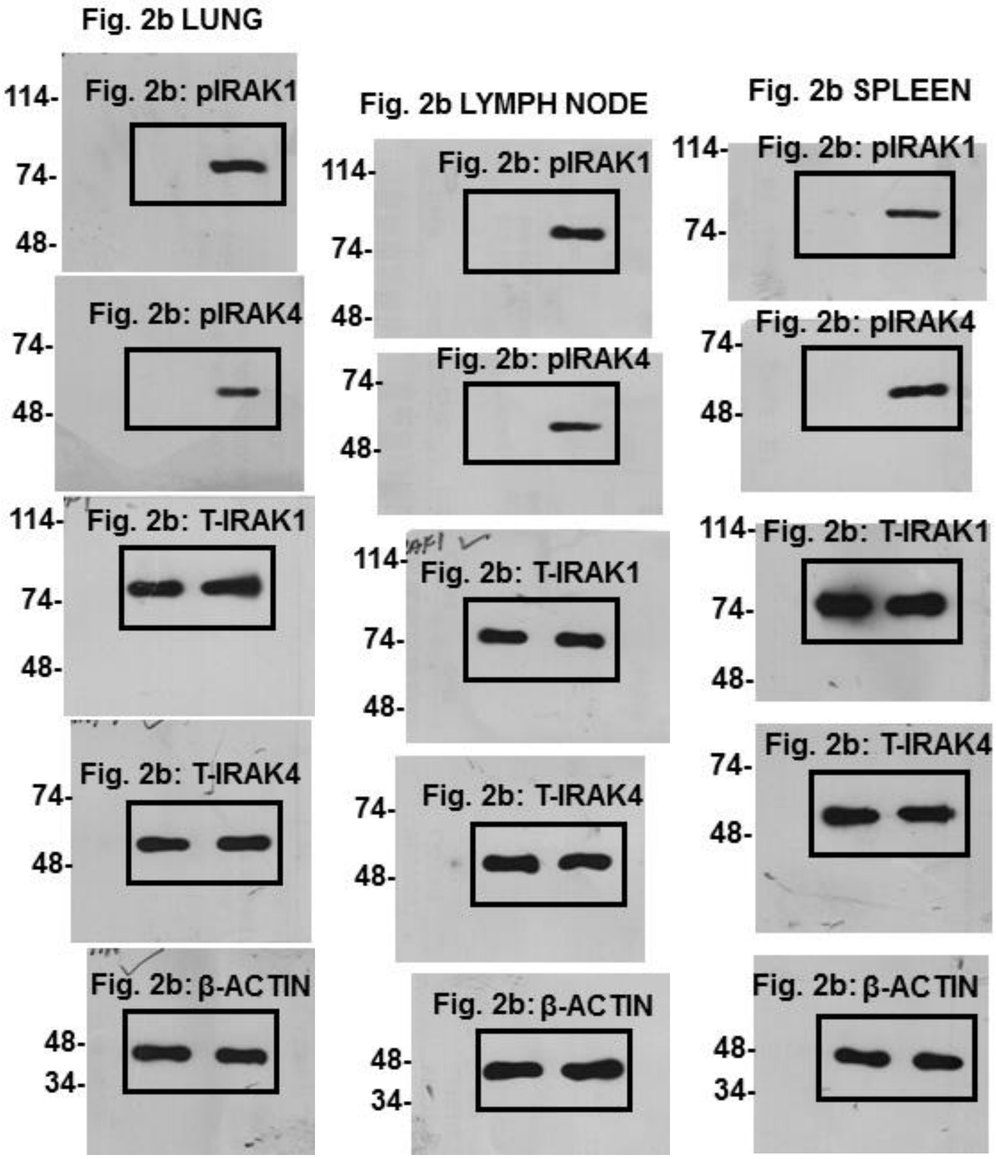

Supplementary Figure S5. Original western blots for images used in Figure 2b. Black boxes indicate the specific bands used in the main figure.

Figure S6

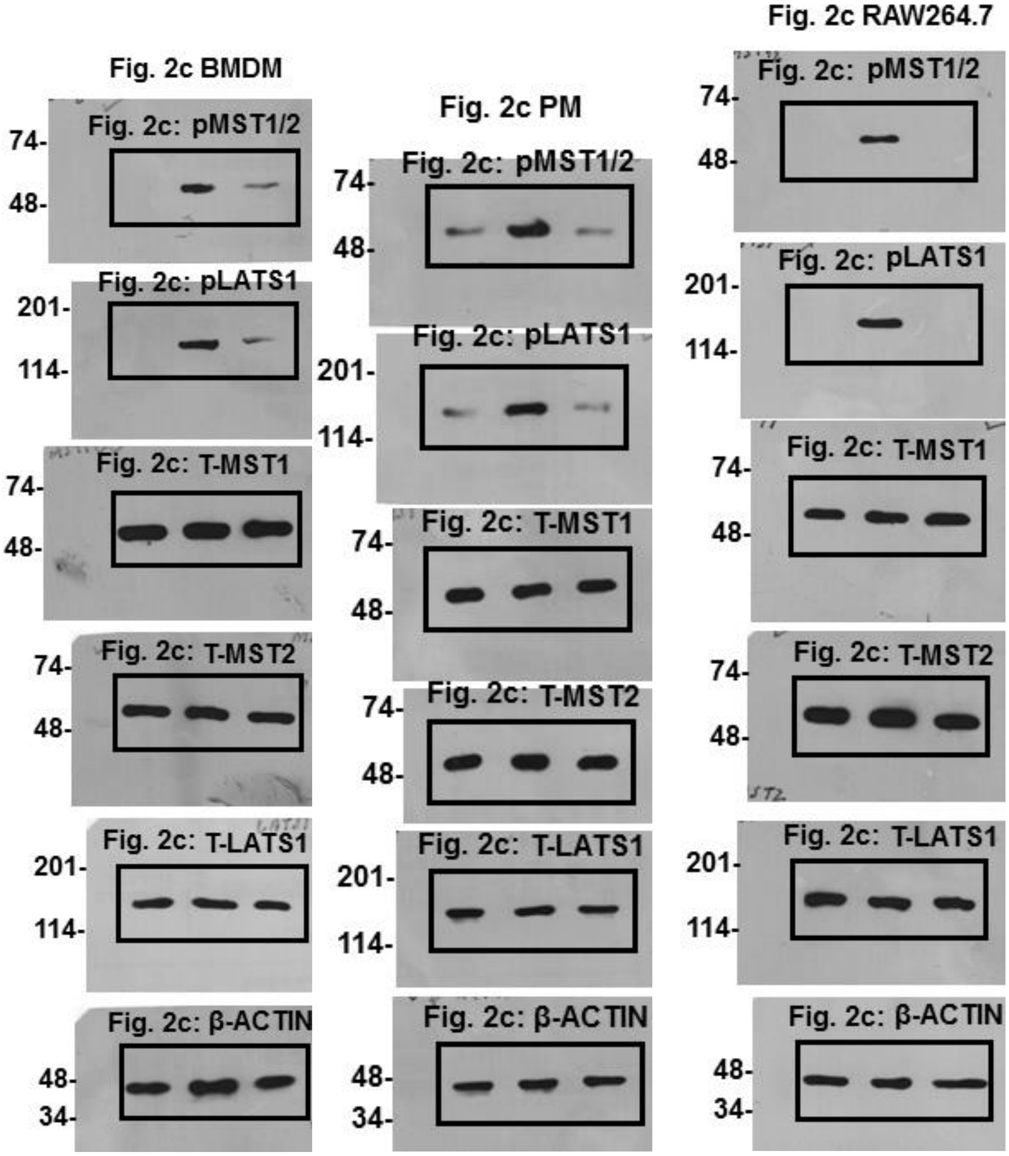

Supplementary Figure S6. Original western blots for images used in Figure 2c. Black boxes indicate the specific bands used in the main figure.

Figure S7

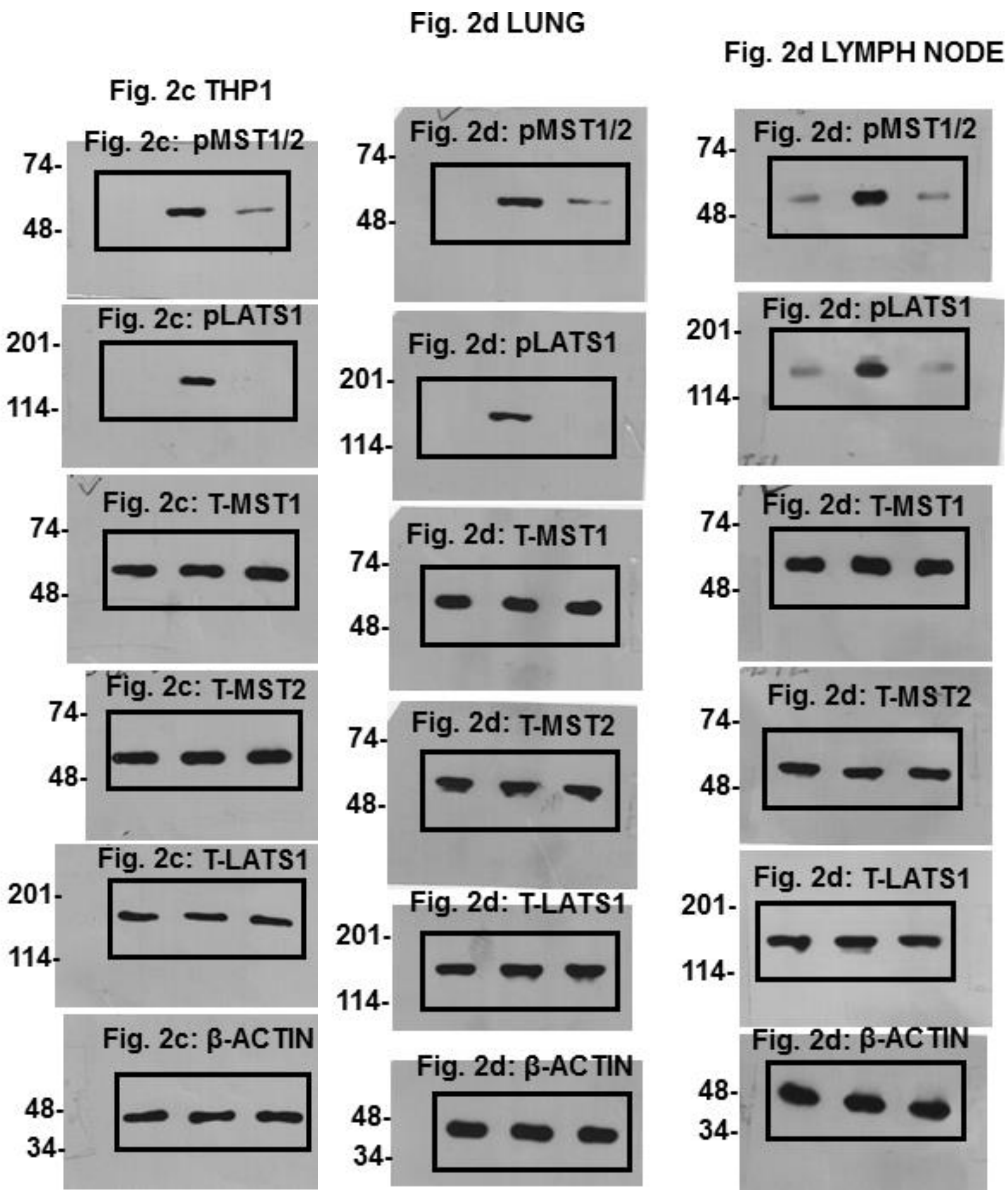

Supplementary Figure S7. Original western blots for images used in Figure 2c (THP1) and 2d (lung and lymph node). Black boxes indicate the specific bands used in the main figure.

Figure S8

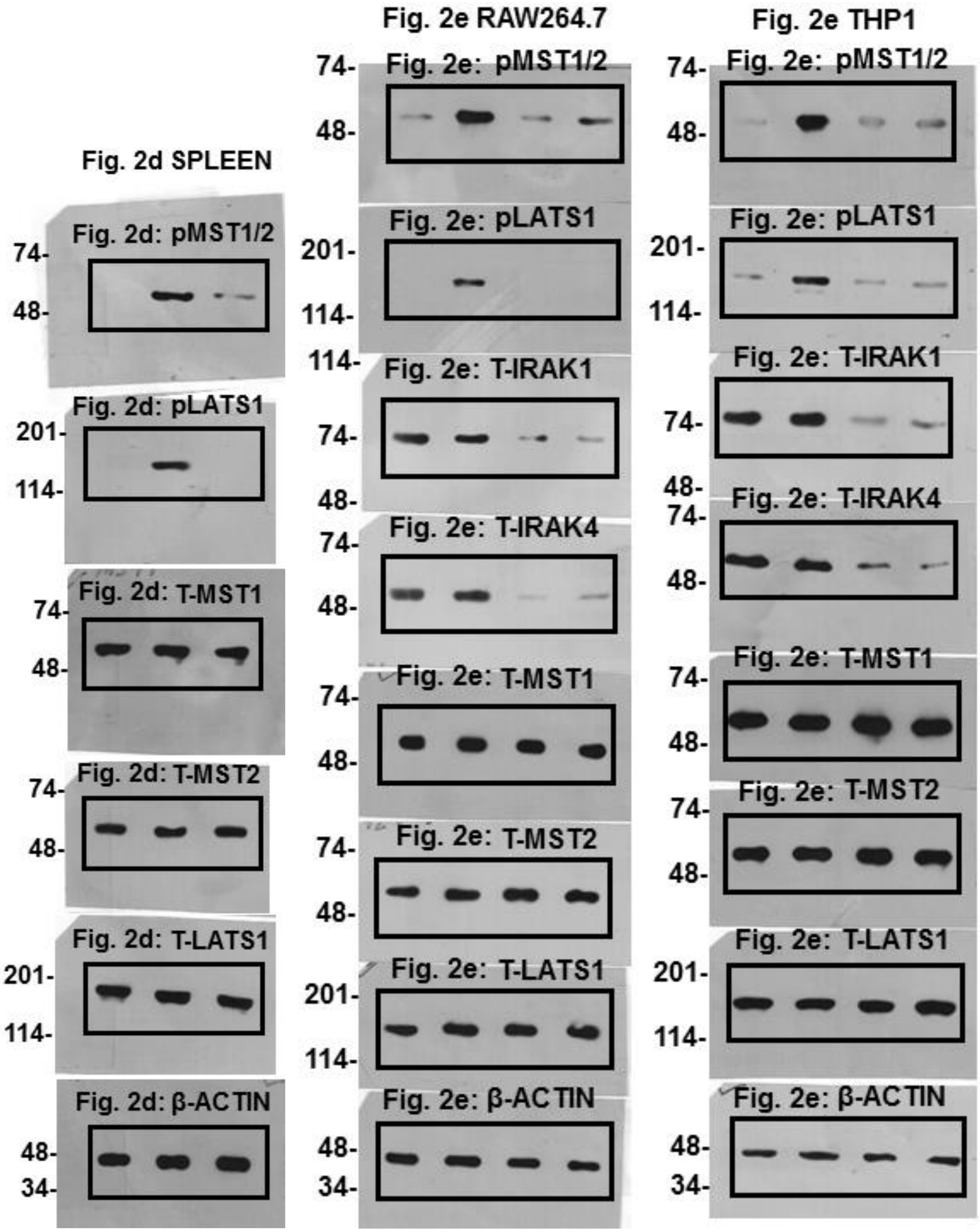

Supplementary Figure S8. Original western blots for images used in Figure 2d (Spleen) and 2e. Black boxes indicate the specific bands used in the main figure.

Figure S9

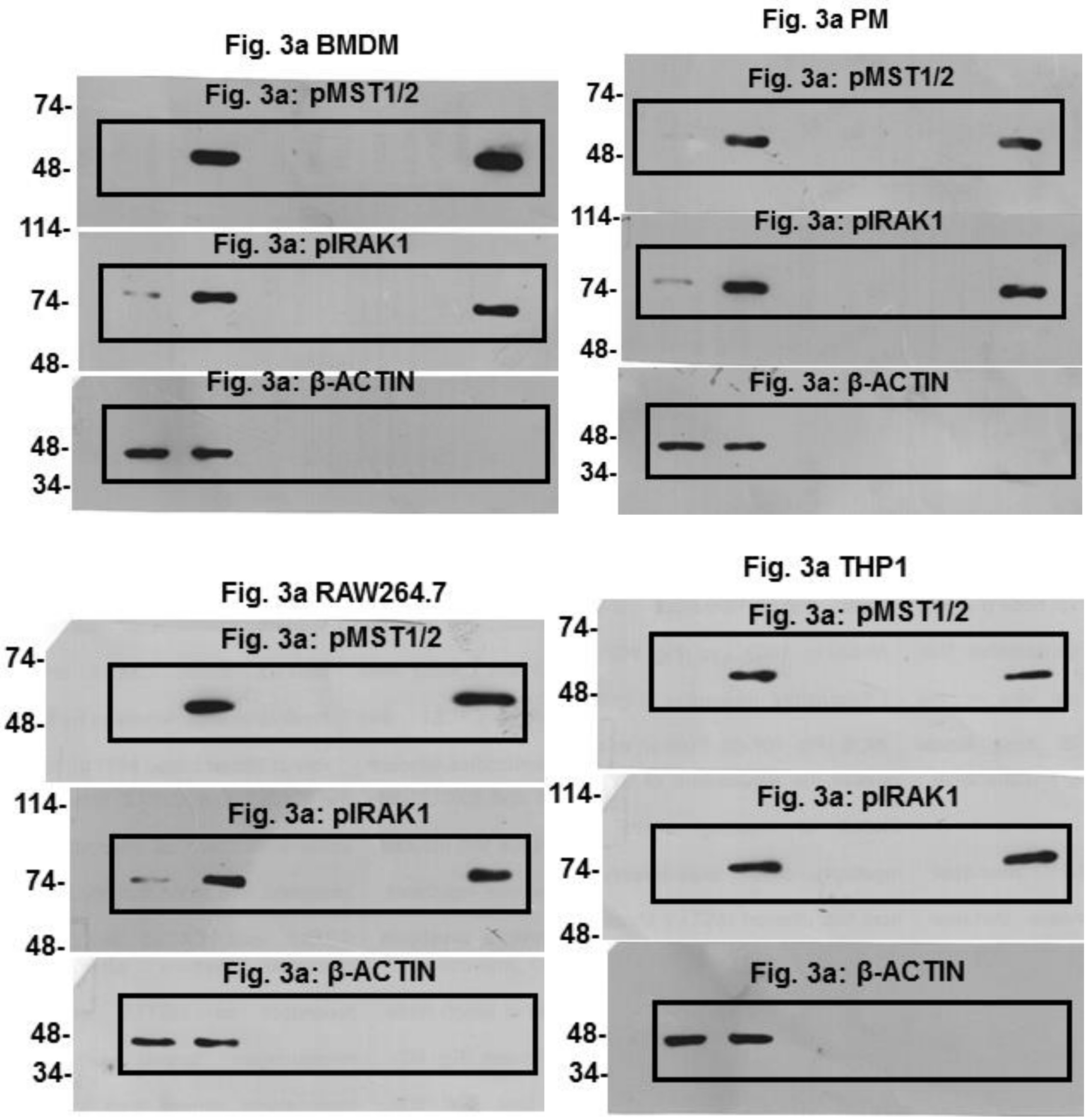

Supplementary Figure S9. Original western blots for images used in Figure 3a. Black boxes indicate the specific bands used in the main figure.

Figure S10

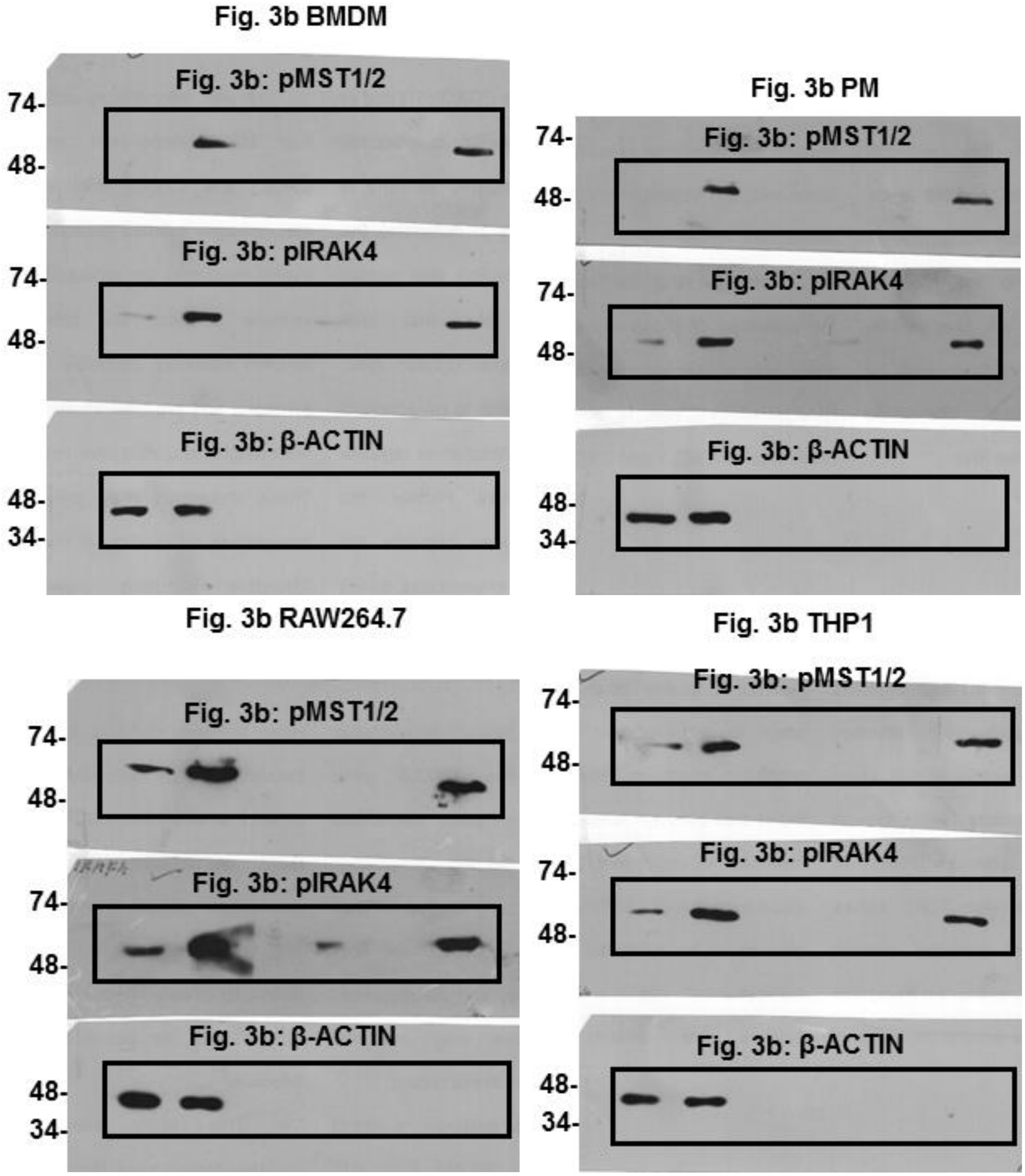

Supplementary Figure S10. Original western blots for images used in Figure 3b. Black boxes indicate the specific bands used in the main figure.

Figure S11

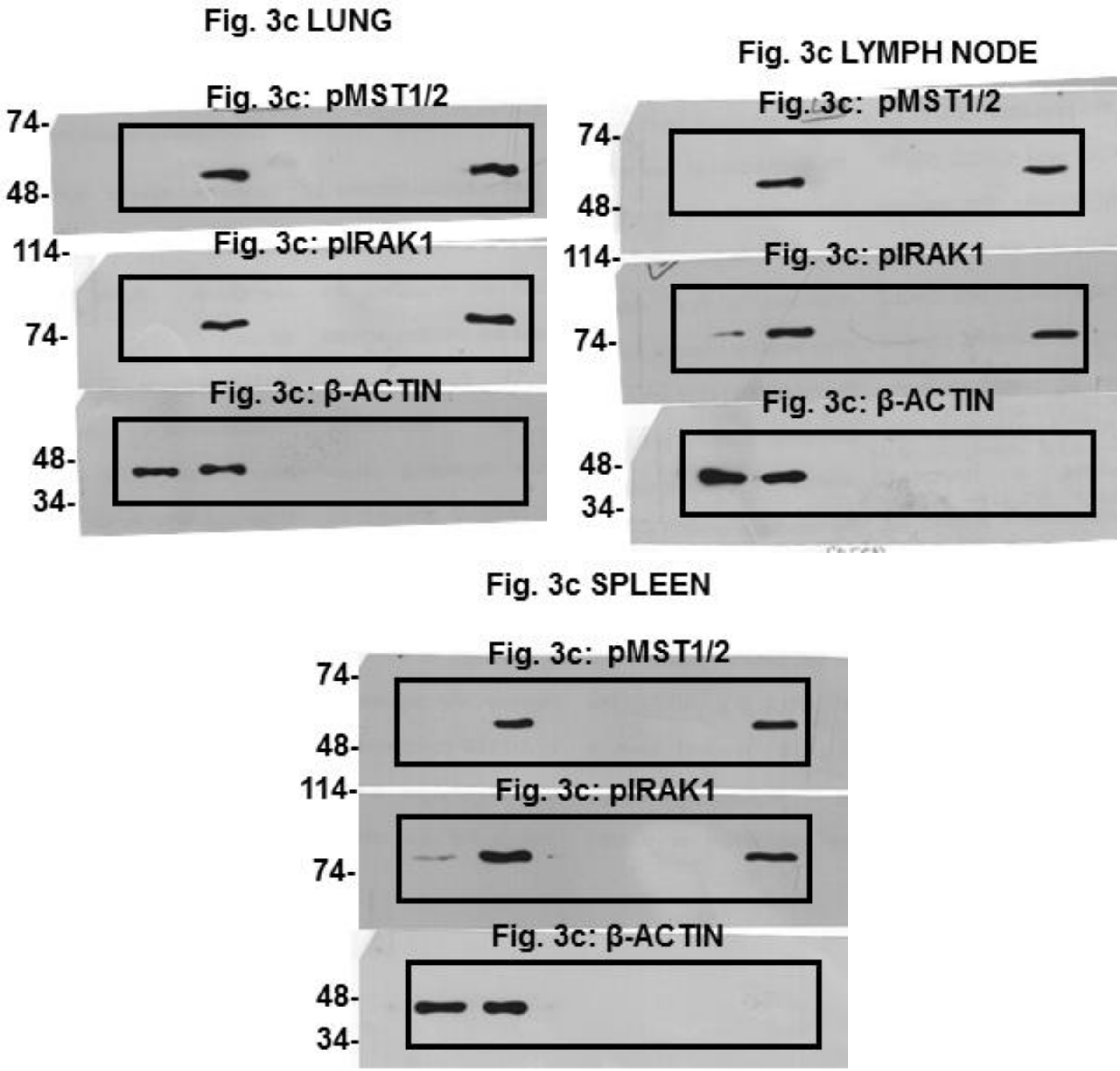

Supplementary Figure S11. Original western blots for images used in Figure 3c. Black boxes indicate the specific bands used in the main figure.

Figure S12

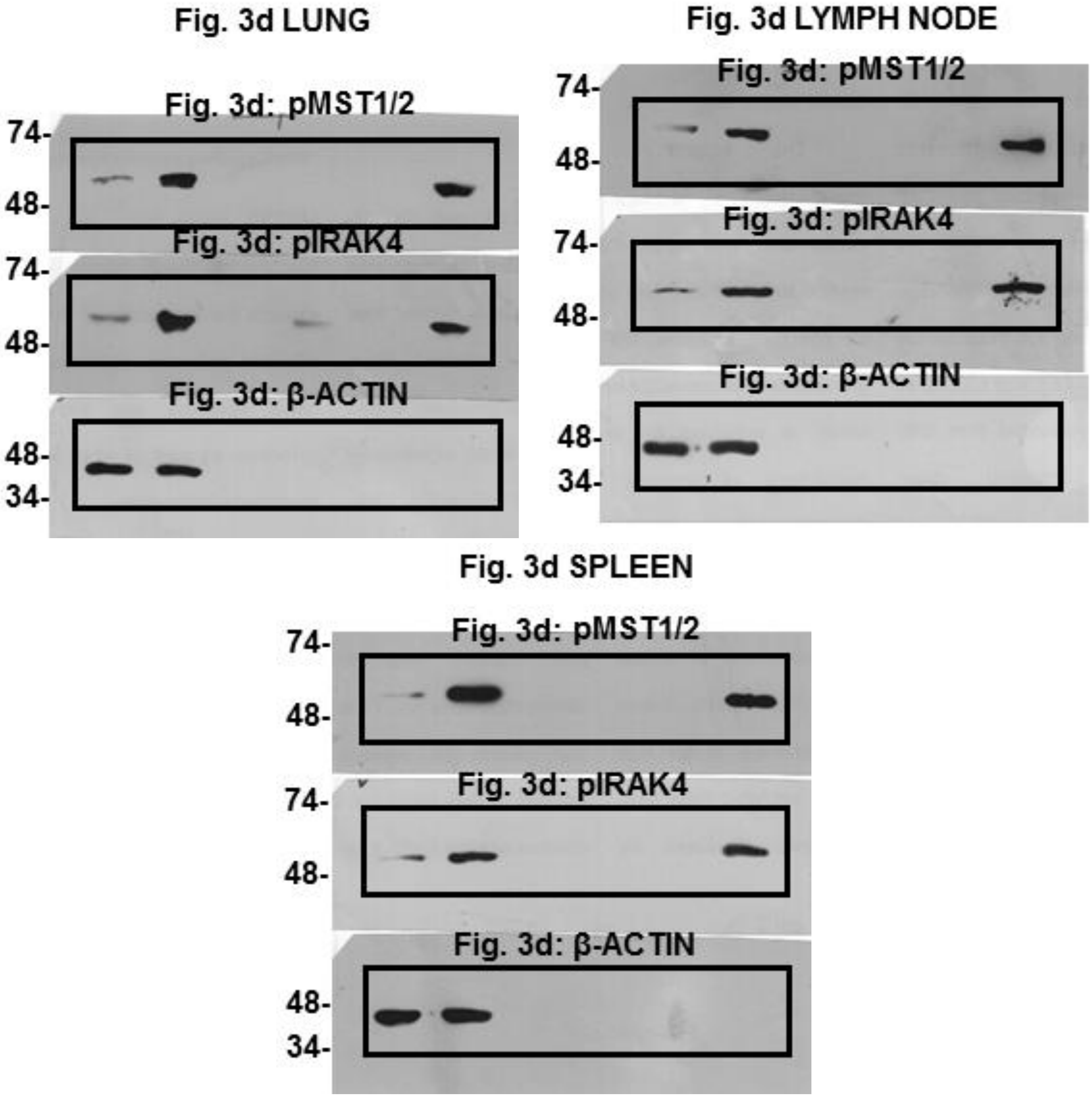

Supplementary Figure S12. Original western blots for images used in Figure 3d. Black boxes indicate the specific bands used in the main figure.

Figure S13

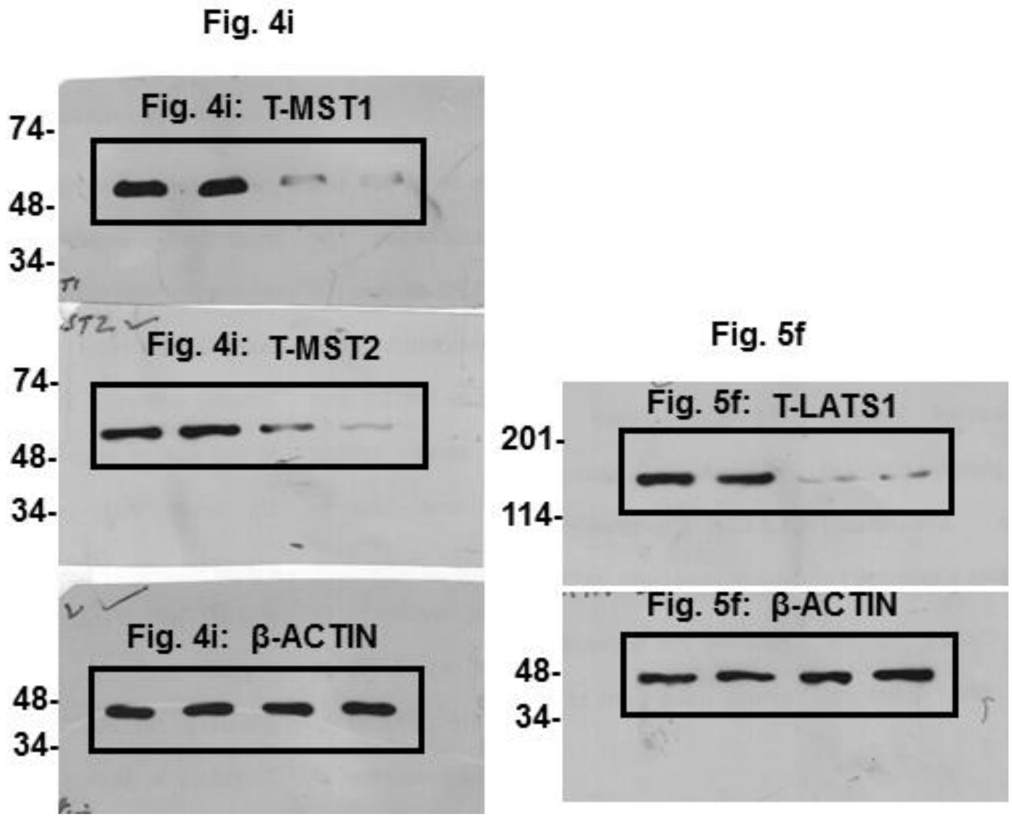

Supplementary Figure S13. Original western blots for images used in Figure 4 and Figure 5. Black boxes indicate the specific bands used in the main figure.

Figure S14

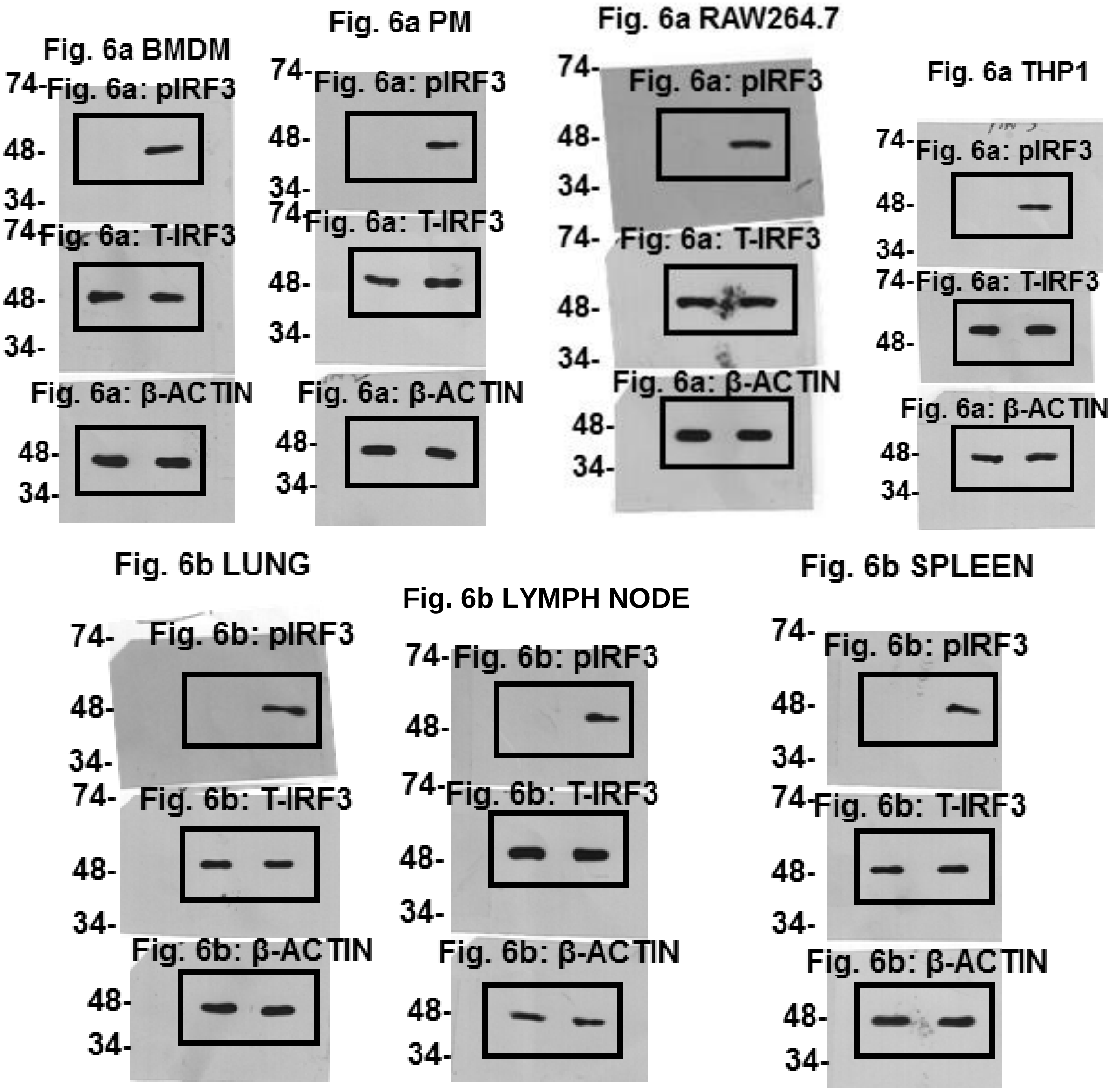

Supplementary Figure S14. Original western blots for images used in Figure 6a and 6b. Black boxes indicate the specific bands used in the main figure.

Figure S15

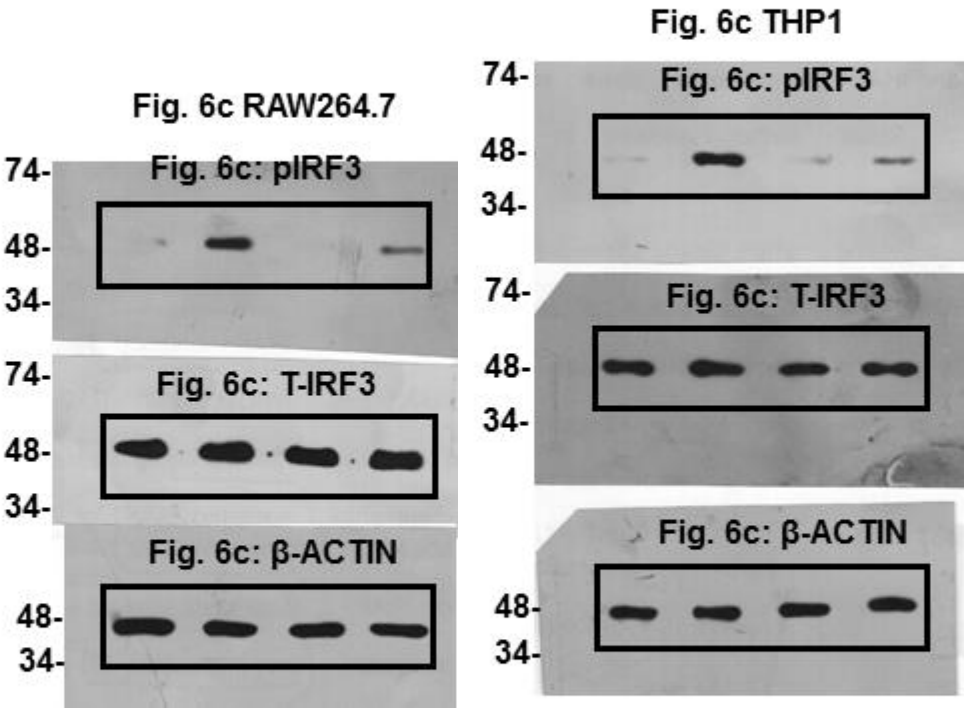

Supplementary Figure S15. Original western blots for images used in Figure 6c. Black boxes indicate the specific bands used in the main figure.

Figure S16

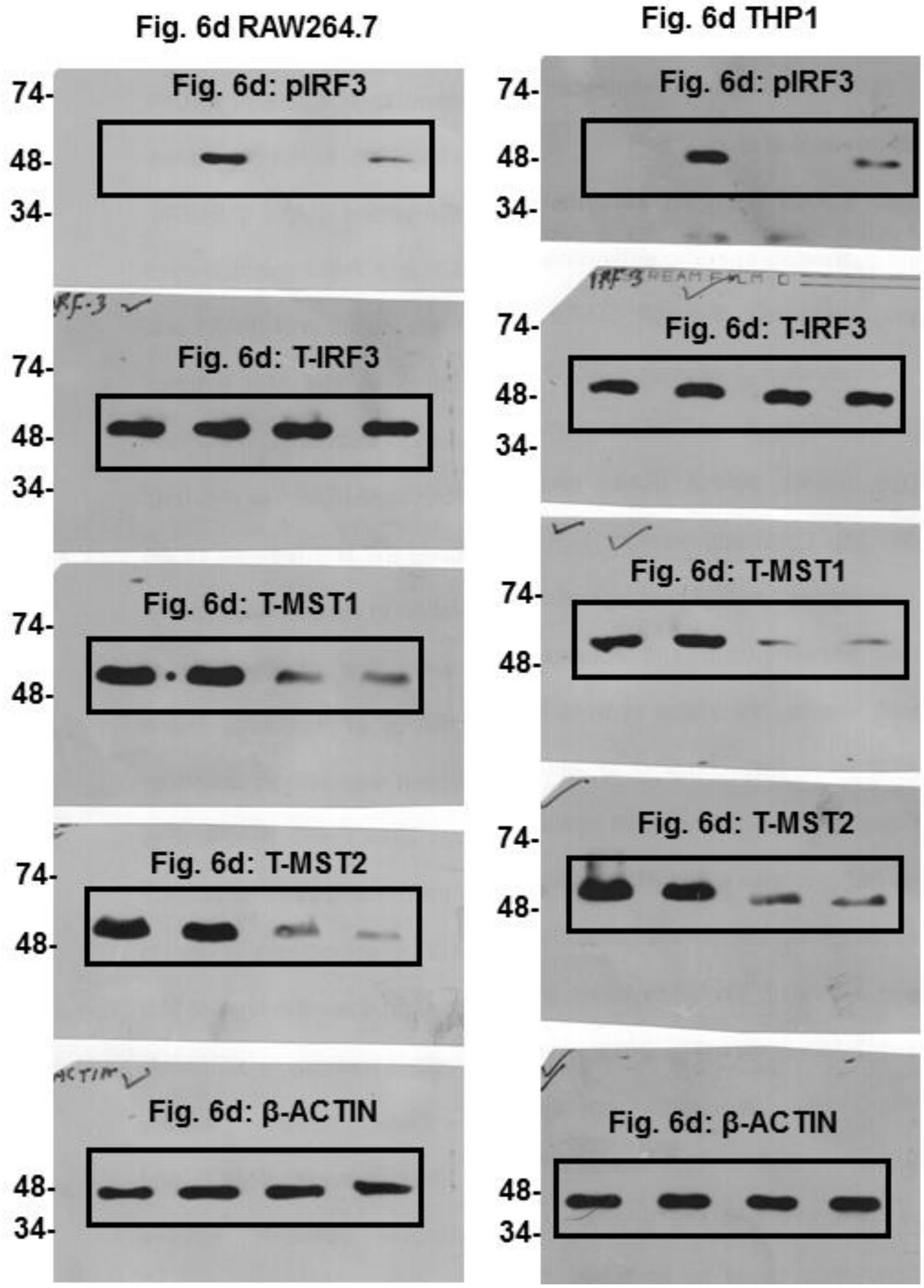

Supplementary Figure S16. Original western blots for images used in Figure 6d. Black boxes indicate the specific bands used in the main figure.

Figure S17

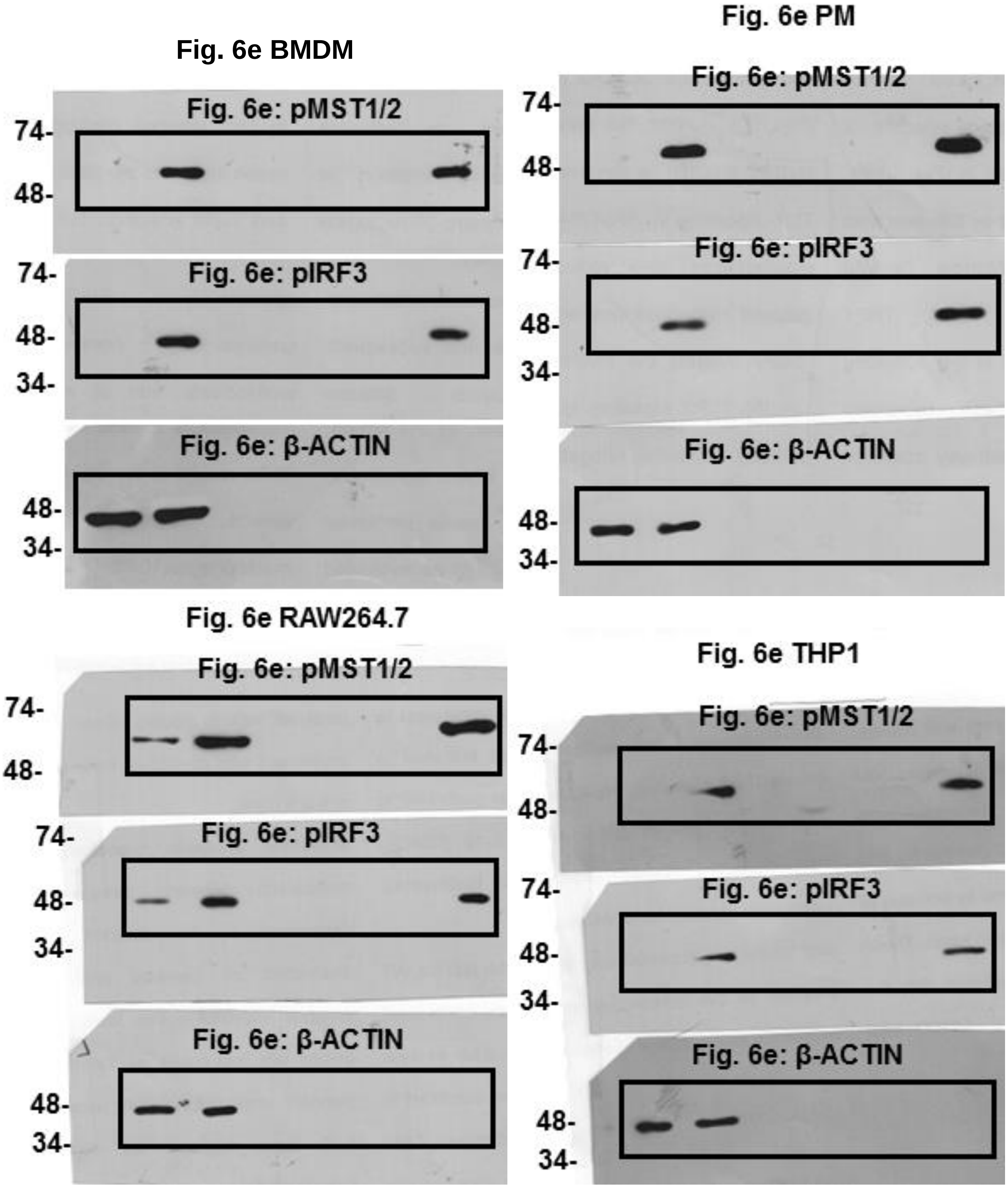

Supplementary Figure S17. Original western blots for images used in Figure 6e. Black boxes indicate the specific bands used in the main figure.

Figure S18

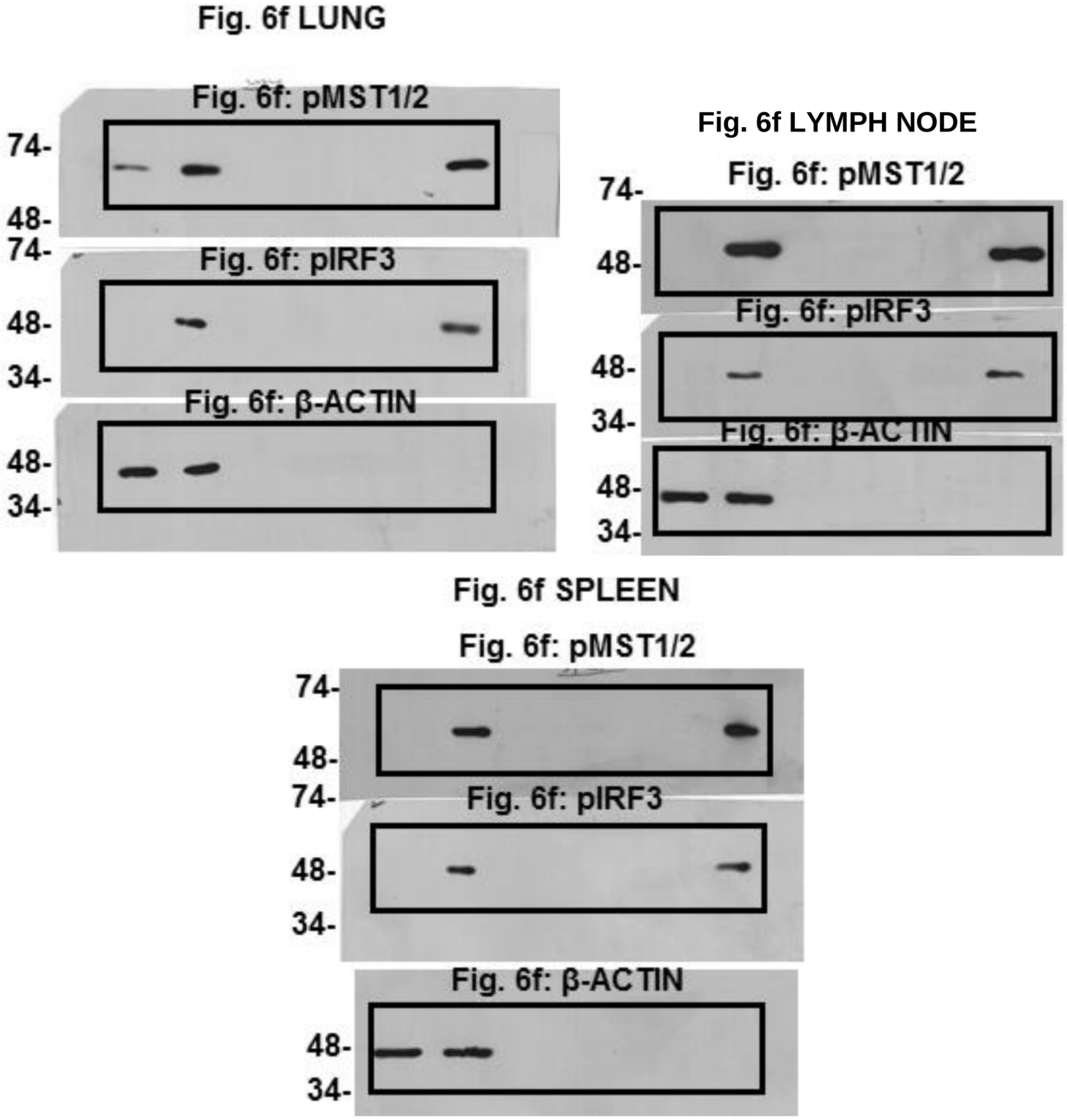

Supplementary Figure S18. Original western blots for images used in Figure 6f. Black boxes indicate the specific bands used in the main figure.

Figure S19

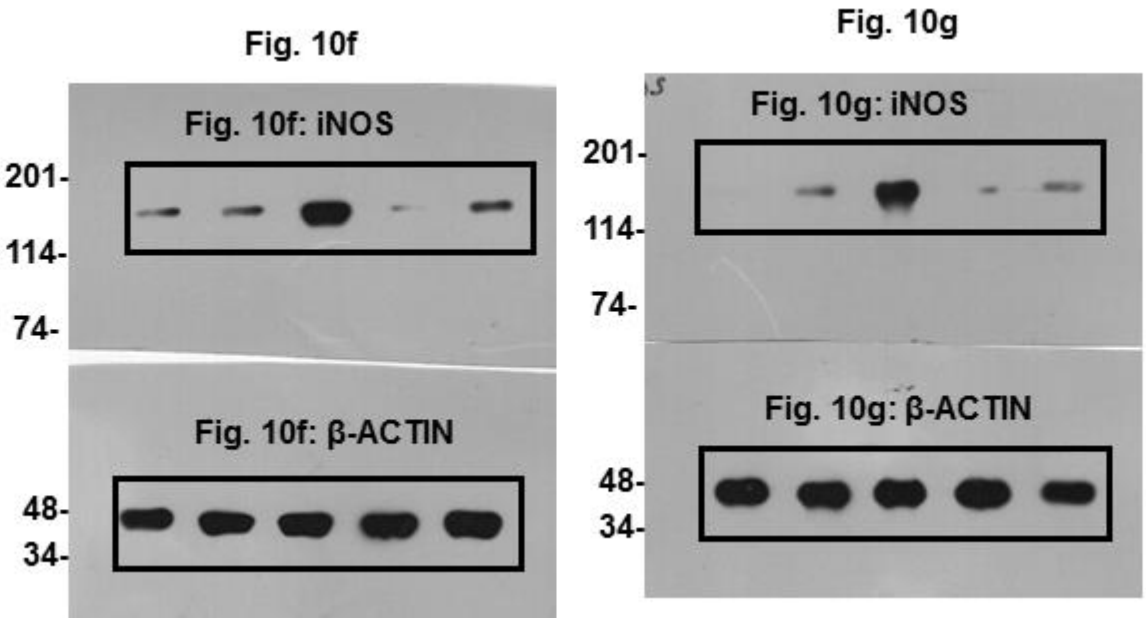

Supplementary Figure S19. Original western blots for images used in Figure 10. Black boxes indicate the specific bands used in the main figure.

Figure S20

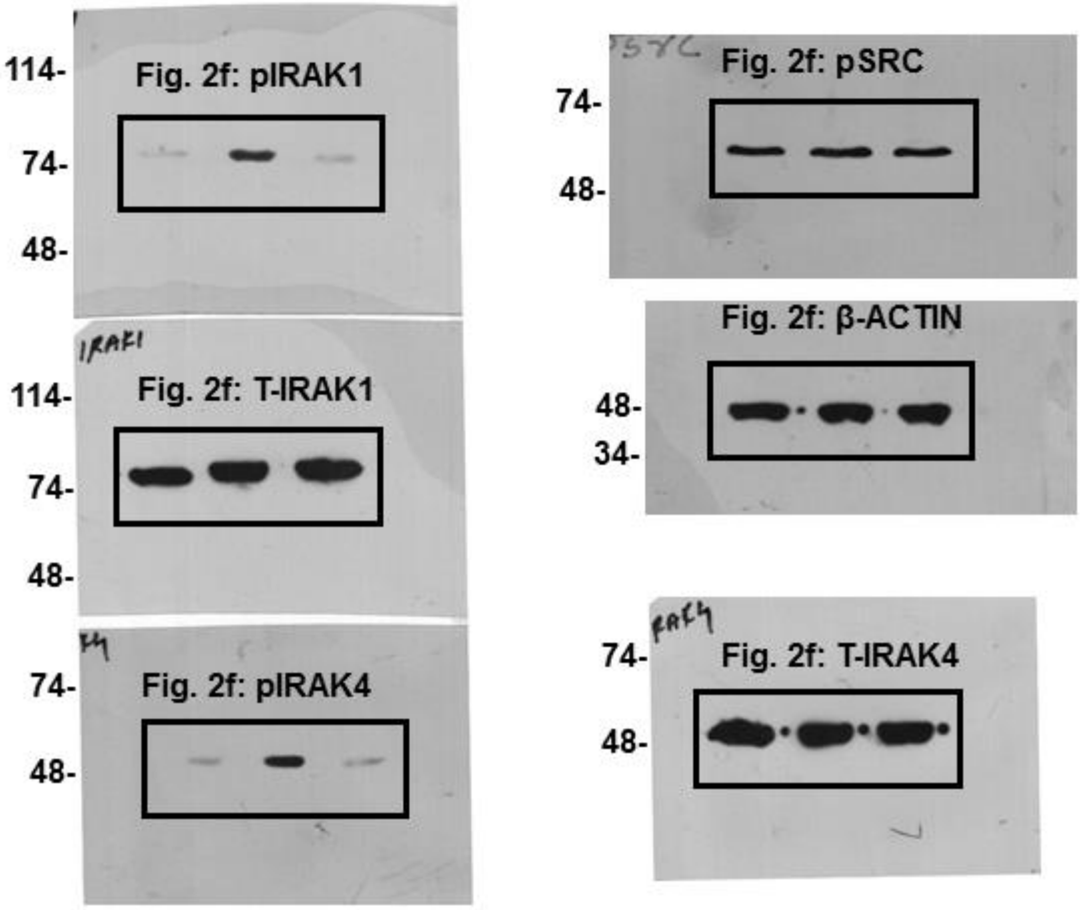

Supplementary Figure S20. Original western blots for images used in Figure 2f. Black boxes indicate the specific bands used in the main figure.

Figure S21

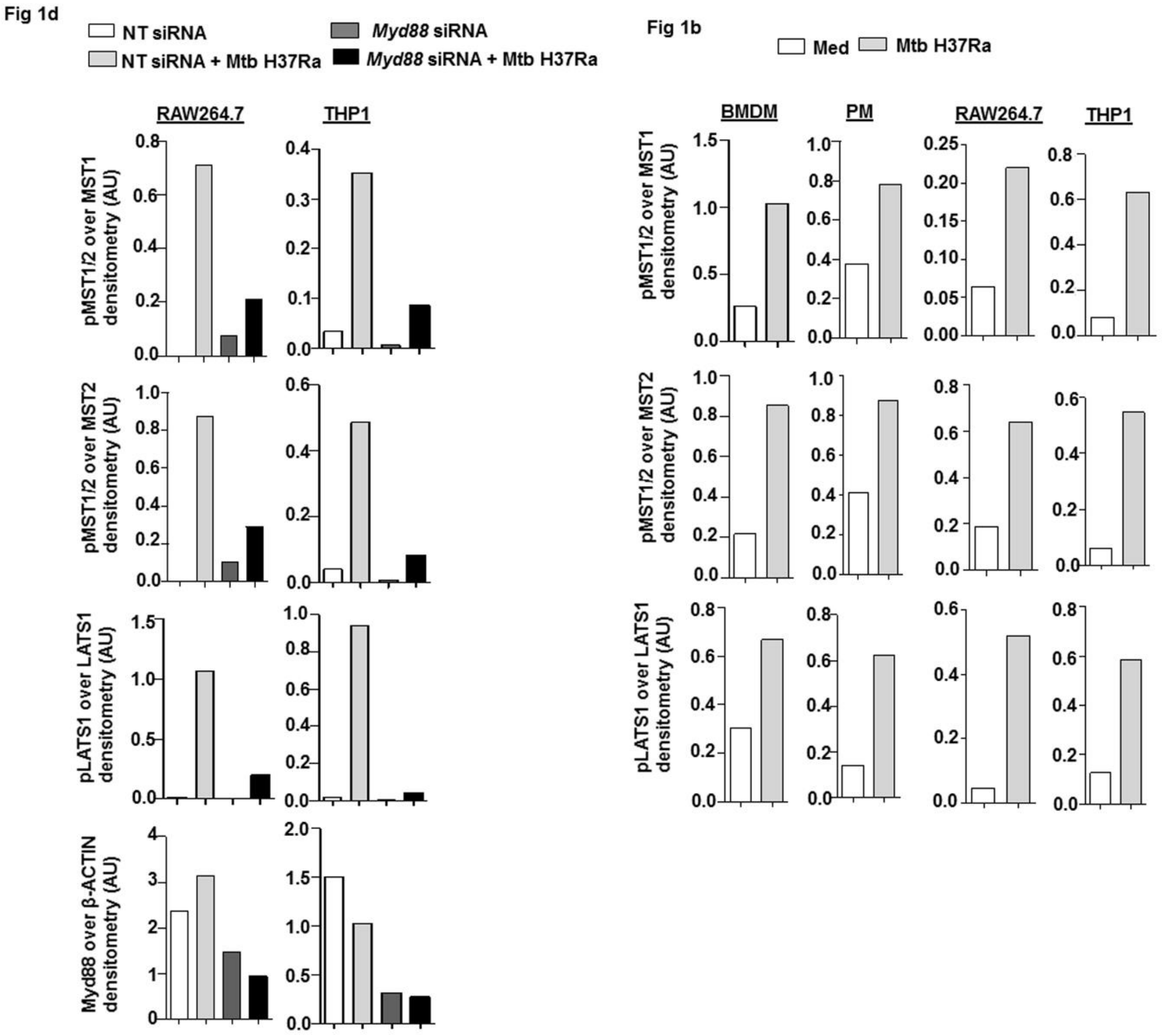

Supplementary Figure S21. Quantitative analysis of western blots for images used in Figure 1b and 1d was performed by using Image Quant TL software.

Figure S22

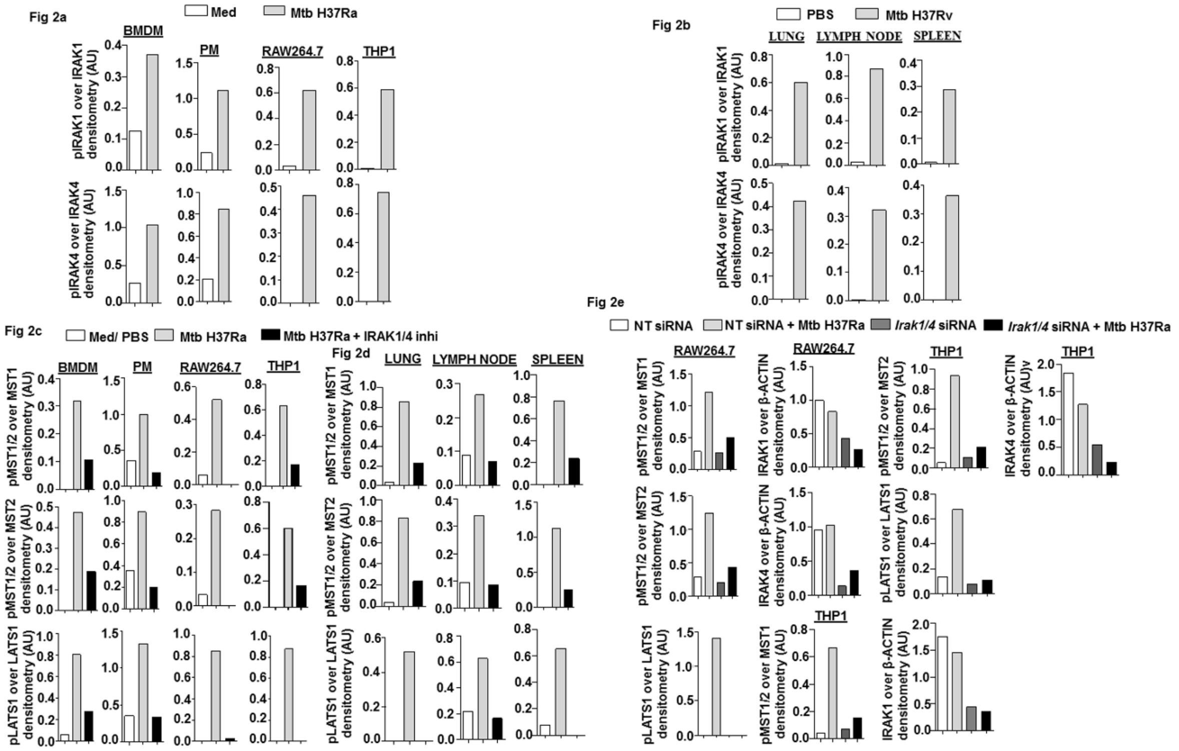

Supplementary Figure S22. Quantitative analysis of western blots for images used in Figure 2a-e was performed by using Image Quant TL software.

Figure S23

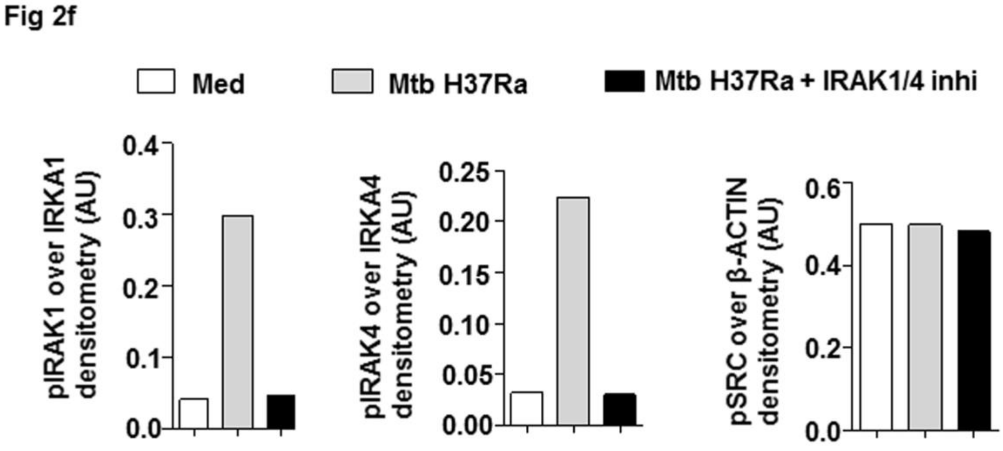

Supplementary Figure S23. Quantitative analysis of western blots for images used in Figure 2f was performed by using Image Quant TL software.

Figure S24

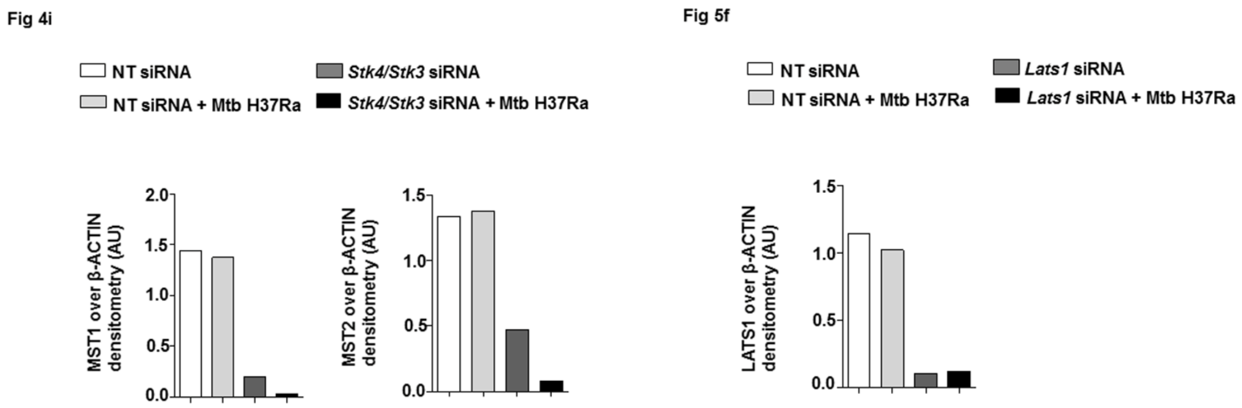

Supplementary Figure S24. Quantitative analysis of western blots for images used in Figure 4i and 5f was performed by using Image Quant TL software.

Figure S25

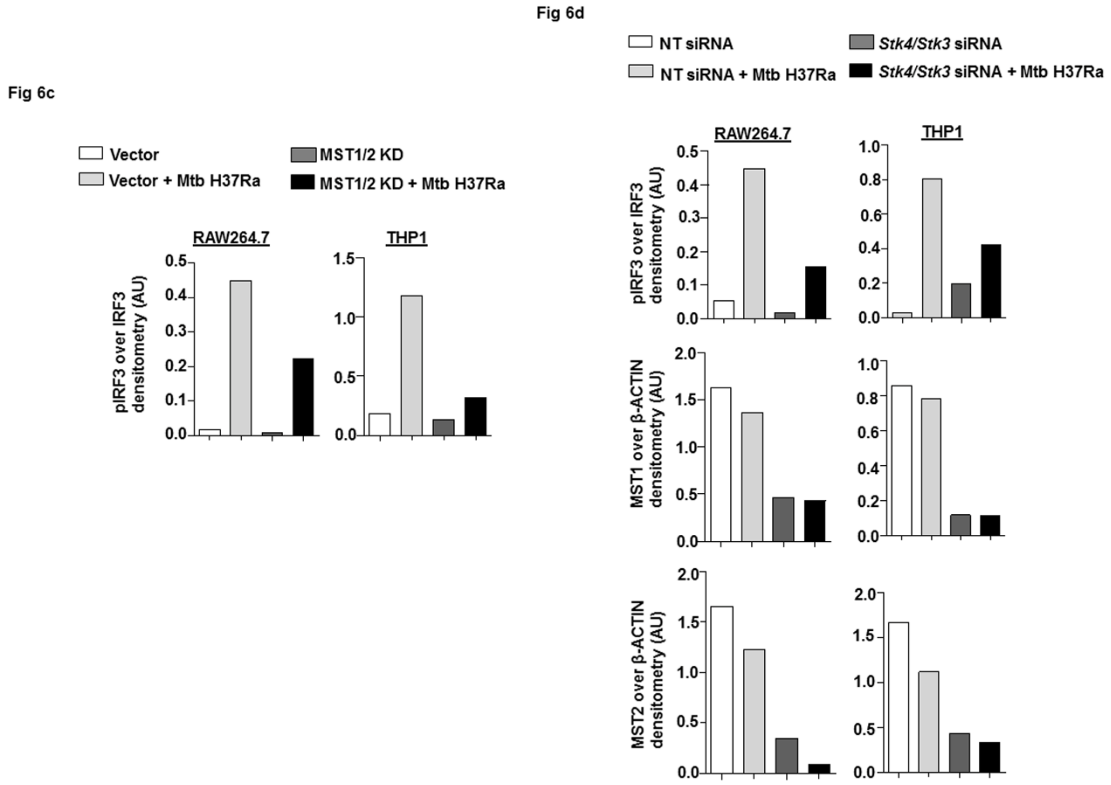

Supplementary Figure S25. Quantitative analysis of western blots for images used in Figure 6c and 6d was performed by using Image Quant TL software.

Figure S26

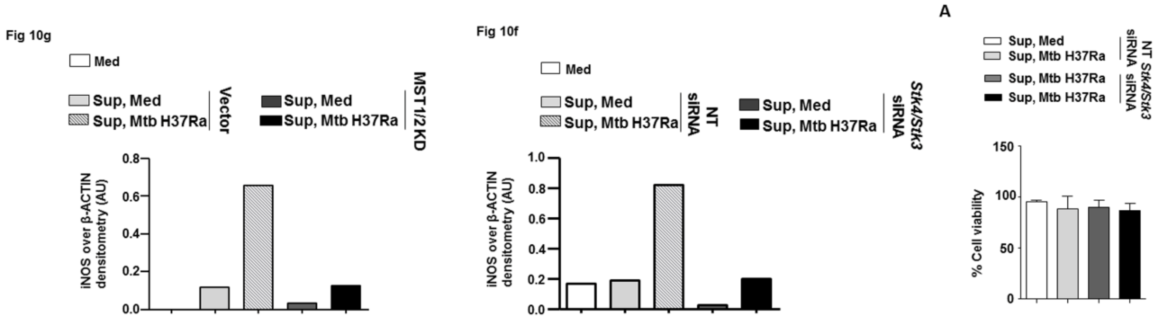

Supplementary Figure S26. Quantitative analysis of western blots for images used in Figure 10g and 10f was performed by using Image Quant TL software. (A) Fresh macrophages were treated with equal volumes of cell culture supernatants derived from MST1/2 knocked down macrophages which were infected for 12 h with Mtb or left uninfected and percentage macrophages cell viability was examined by MTT assay.

Figure S27

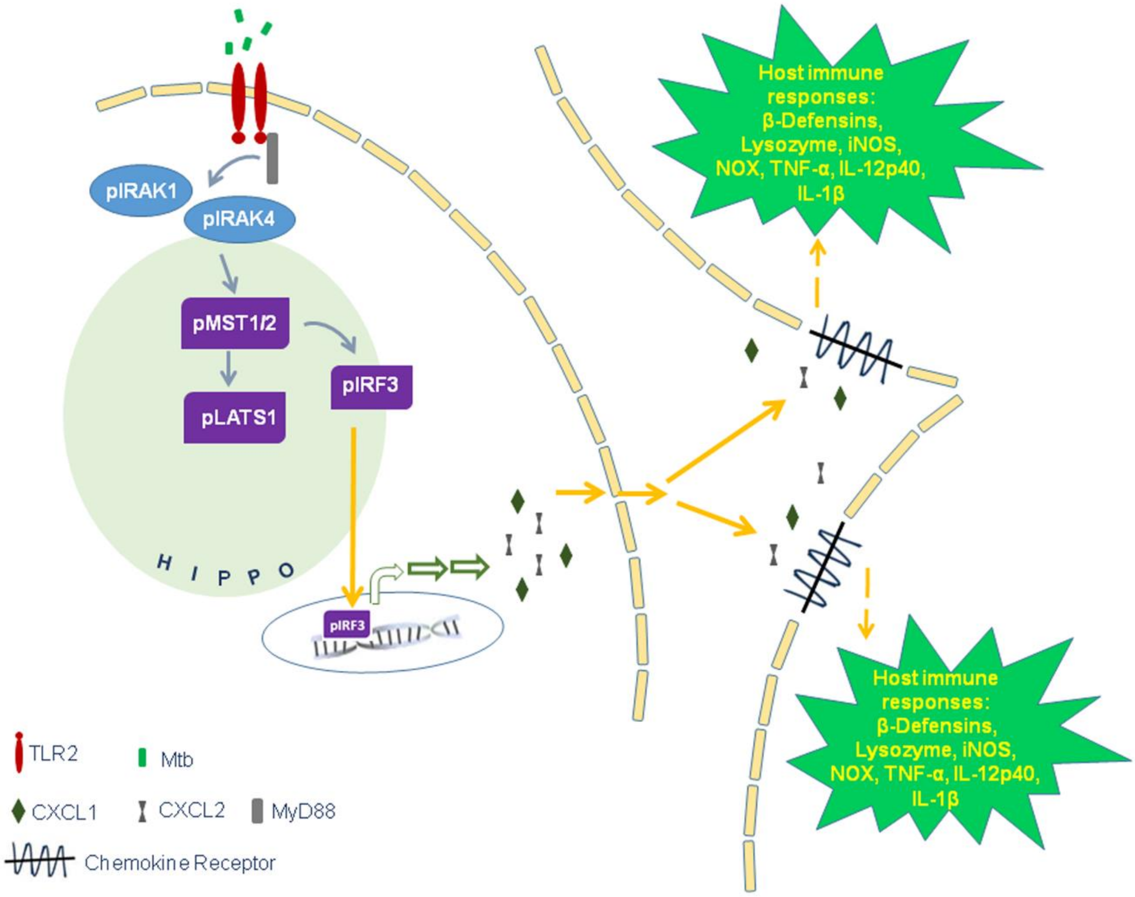

Supplementary Figure S27. Model. Infection of macrophages with Mtb leads to the activation of Hippo signaling pathway in a TLR2-dependent manner through IRAK1 and IRAK4. Hippo (MST1/2 in mammals) regulates the production of chemokines CXCL1 and CXCL2 by activating a non-canonical MST1/2 effector, IRF3, during mycobacterial pathogenesis. CXCL1 and CXCL2 in turn act on neighboring cells in a paracrine fashion to induce the expression of innate immune mediators like pro-inflammatory cytokines and beta-defensins.
